# Dynamic machine learning architecture integrating time-phased predictions for acute kidney injury following extracorporeal membrane oxygenation

**DOI:** 10.3389/fphys.2026.1922656

**Published:** 2026-10-22

**Authors:** Jianhai Lu, Zhen Chen, Donghua Zheng, Genglong Liu, Yijia Wu, Wanfang Li, Liuer Zuo, Qiang Tai

**Affiliations:** 1 Department of Intensive Care Unit, The Eighth Affiliated Hospital, Southern Medical University (The First People’s Hospital of Shunde, Foshan), Foshan, Guangdong, China; 2 Department of Critical Care Medicine, The First Affiliated Hospital of Sun Yat-sen University, Guangzhou, Guangdong, China; 3 School of Medicine, Southern Medical University, Foshan, Guangdong, China; 4 Editor Office, iMeta, Shenzhen, Guangdong, China

**Keywords:** acute kidney injury, dynamic prediction model, extracorporeal membrane oxygenation, machine learning approaches, model interpretability

## Abstract

**Background:**

Acute kidney injury (AKI) is a frequent, severe complication of extracorporeal membrane oxygenation (ECMO) therapy, substantially contributing to unfavorable outcomes. No effective machine learning (ML) models exist for early, accurate detection. This study developed a two-phased prediction model using clinical data from pre-ECMO and on-ECMO stages for ECMO-induced AKI (ECMO-AKI), and further integrated two models to formulate a consensus risk-stratification strategy.

**Methods:**

This retrospective study included 344 patients undergoing ECMO from 2015 to 2026. Clinical and laboratory variables from pre-ECMO and on-ECMO stages were respectively used to develop two ECMO-AKI diagnostic models via a analytical framework incorporating 12 ML algorithms. Multiple analytical methods were utilised to evaluate pre-ECMOAKI and on-ECMOAKI models. The Shapley Additive Explanations (SHAP) method was used to interpret two models and to quantify and visualize the features. Latent class growth mixture model (LCGMM) was applied to investigate the association between longitudinal feature trajectories and ECMO-AKI.

**Results:**

Pre-ECMOAKI and on-ECMOAKI models incorporated into 12 ML, yielding 66 and 91 combinations, respectively. Among these, glmBoost + gradient boosting machine (GBM) as a superior algorithm for predicting ECMO-AKI. Pre-ECMOAKI (9 features) and on-ECMOAKI (10 features) models exhibited superior discrimination performance, with area under curve (AUC) of 0.867 (95%CI: 0.819-0.911) and 0.907 (95%CI: 0.870-0.941) in derivation cohort, 0.732 (95%CI: 0.678-0.780) and 0.803 (95%CI: 0.711-0.889) in held-out validation cohort, respectively. A risk-stratification framework integrating pre-ECMOAKI and on-ECMOAKI predictions achieved a class-specific precision of 90.23% for ECMO-AKI events in the consensus high-risk group and 90.79% for non-ECMO-AKI events in the consensus low-risk populations. SHAP analysis reveals that endothelial activation and stress index (EASIX), cardiopulmonary resuscitation time (CPRT) and total bilirubin (TBI) are important feature variables in two models. Restricted cubic spline (RCS) regression, threshold effect analysis, and LCGMM indicated EASIX > 4.7 (with sustained increasing trend), CRPT > 24 min, and TBI > 20.2 μmol/L (with continuous upward trend) were associated with an elevated ECMO-AKI risk.

**Conclusion:**

Two-phased dynamic prediction models (pre-ECMOAKI and on-ECMOAKI) were constructed based on glmBoost +GBM, which act as an accurate and interpretable diagnostic tool for ECMO-AKI. Based on consistency analysis of predictions of two models, this framework allows early risk stratification and thus holds potential for individualized management.

## Introduction

Extracorporeal membrane oxygenation (ECMO) acts as last-line support for severe reversible cardiopulmonary dysfunction unresponsive to standard therapy to sustain organ perfusion and oxygen supply (Hadaya and Benharash, 2020). Global ECMO usage surged amid the COVID-19 pandemic, verifying its efficacy for resuscitating critically ill patients (Tonna et al., 2024). Despite advancements in ECMO technology and management, mortality following ECMO remains substantial—ranging from 44% to 62% (Feistritzer et al., 2025). Of these, acute kidney injury (AKI) stands as a typical complication in patients undergoing ECMO, with an overall incidence of 50%-70% regardless of age groups, which is a pivotal contributor to elevated mortality, extended hospitalization, and rising medical expenses (Guru et al., 2024). In other clinical settings, structured protocols integrating risk assessment tools, early AKI detection and monitoring, as well as standardized interventions have achieved a 20%-30% reduction in AKI incidence (Göcze et al., 2018; Zarbock et al., 2021). Accordingly, accurate and timely identification of high-risk ECMO-supported patients creates a critical window for proactive management, which is essential to prevent ECMO-AKI.

In routine clinical practice, AKI is diagnosed via increased serum creatinine (Cr) and reduced urine output (UO) following the Kidney Disease: Improving Global Outcomes (KDIGO) criteria (Kidney Disease: Improving Global Outcomes (KDIGO) Acute Kidney Injury Work Group, 2012), yet these biomarkers exhibit unsatisfactory diagnostic sensitivity and specificity (Ronco et al., 2019). Cr cannot detect impending AKI, as its elevation emerges subsequent to established kidney injury, restricting timely therapeutic intervention. The pathophysiology of ECMO-AKI is multifactorial, involving insufficient renal perfusion, ischemia-reperfusion injury, endothelial activation and injury, activation of inflammatory cascades, oxidative stress, nephrotoxin exposure, and other mechanisms (Ostermann and Lumlertgul, 2021). These factors interact synergistically to collectively drive the initiation and progression of ECMO-AKI. Several prior studies have investigated risk factors for ECMO-AKI and identified hemoglobin, lactate, sequential organ failure assessment (SOFA), left ventricular ejection fraction, and acute physiology and chronic health evaluation II (APACHE II) as its independent risk predictors (Lepère et al., 2020; Mou et al., 2022; Chen et al., 2023). Nevertheless, such isolated static indicators cannot reflect real-time dynamic alterations in renal injury and recovery among ECMO recipients. To date, no well-established time-phased risk assessment models enable early and precise evaluation of ECMO-AKI risk, hindering individualized optimization of ECMO clinical strategies.

Machine learning (ML) holds substantial potential in healthcare (van de Sande et al., 2021). Following algorithm development and implementation, ML models can learn from sample data and historical cohorts, uncover concealed variable associations in big datasets, and complete predictive and risk classification analyses based on baseline indicators (Ge et al., 2025). In recent years, ML models developed from electronic medical records (EMRs) have garnered widespread attention and recognition among clinicians (Chen Z. et al., 2026). The advent of EHRs, comprehensive monitoring, and real-time data availability, combined with advanced data analytics, has facilitated the development of time-phased ML models (Lapp et al., 2023).

In this retrospective cohort study, clinical information were systematically collected from patients during pre-ECMO and on-ECMO periods. Subsequently, we formulated a multi-criteria decision-making system by converting 12 classical ML algorithms into multiple combinations, from which accurate and robust ML models were selected for developing the two-stage ECMO-AKI prediction model. Finally, patients were stratified by risk levels based on the predictive outputs of the pre-ECMOAKI and on-ECMOAKI models. A risk stratification strategy relying on prediction consistency between the two models was proposed to boost the overall predictive performance.

## Materials and methods

### Study design

This study adopted a time-phased predictive architecture consisting of two independent models: (1) a pre-ECMO model built on variables collected within 24 hours prior to ECMO cannulation and initiation; and (2) an on-ECMO model incorporating parameters measured within the initial 4 hours of ECMO implementation. Each model was individually trained and tuned using dataset exclusive to its corresponding time interval, thereby eliminating cross-time-window information leakage. The overall research workflow was divided into seven modules: Data acquisition and preprocessing, model development and validation, model evaluation, online model calculator, model interpretation, model collaborative prediction framework, and model feature analysis. This study followed the STROBE (Cuschieri, 2019) and TRIPOD + artificial intelligence (AI) guidelines (Collins et al., 2024), with the flowchart illustrated in **Figure 1**.

**Figure 1:**
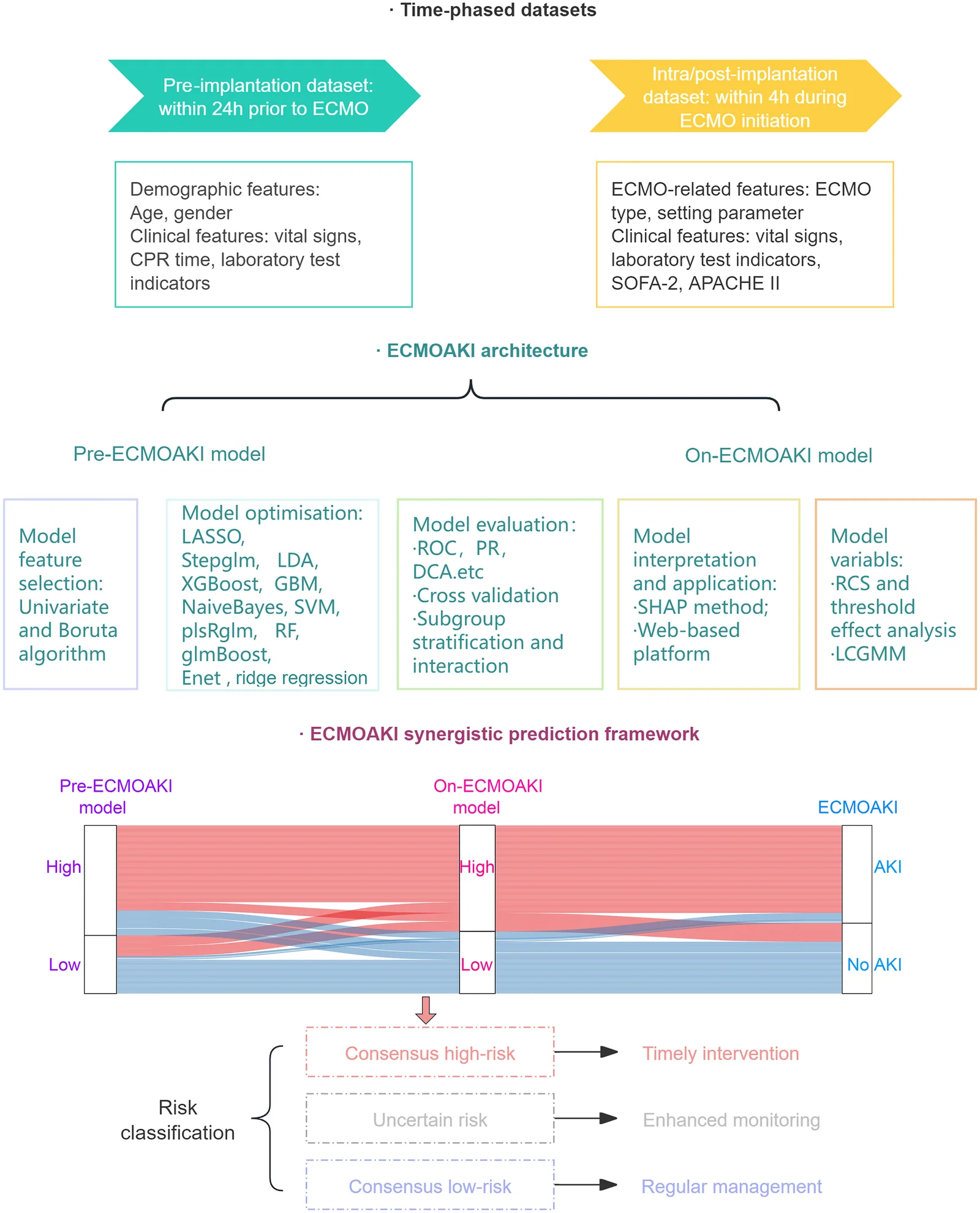
Flowchart illustrating the study design and analytical procedures in this cohort study.

### Study setting and participants

This retrospective cohort study enrolled 344 patients who received ECMO support in the Intensive Care Unit (ICU) of the Eighth Affiliated Hospital, Southern Medical University, between January 2015 and April 2026. Extracorporeal Life Support Organization (ELSO) guidelines (Ali and Vuylsteke, 2019) were adopted to define ECMO implantation indications.

Inclusion criteria: (1) Age ≥ 18 years, and (2) ECMO support duration ≥ 24 hours. Exclusion criteria: (1) Preexisting stage 5 chronic kidney disease (CKD) or established AKI; (2) Prior administration of renal replacement therapy (RRT); (3) Overall missing data proportion exceeding 15%; (4) Mortality within 24 hours after ECMO initiation.

### Sample size determination

Sample size calculation was performed according to the events-per-variable (EPV) rule. For the planned 5 to 10 candidate predictors, a minimum of 50 to 100 AKI events was needed. Given the institutional retrospective data indicating an AKI incidence rate of approximately 60% among ECMO recipients, the patients were estimated to range from 84 to 167. Accounting for 10% potential data loss, the target sample size for the derivation cohort was revised to 94 to 186 patients. Finally, 344 ECMO-supported patients were enrolled and randomly split into a derivation cohort (238 patients) and a validation cohort (106 patients) at a 7:3 ratio, which exceeded the calculated requirement.

### Data collection

Based on the timing of data collection, variables were categorised as follows: demographic data, past medical history, clinical characteristics, and laboratory measurements obtained within 24 hours before ECMO cannulation were defined as pre-ECMO variables; parameters pertaining to ECMO setup, together with clinical characteristics and laboratory assays acquired within the initial 4 hours after ECMO initiation, were classified as on-ECMO variables. Furthermore, 24-hour severity scores (SOFA-2 (Ranzani et al., 2025) and APACHE II) and fluid management (input/output volume and UO) post ECMO commencement, concomitant treatments and medications during ECMO, and clinical endpoints (ECMO runtime, mechanical ventilation (MV) duration, mechanical ventilation (MV) time, ECMO treatment time, ICU/hospital length of stay (LOS), and survival to discharge) were recorded.

The endothelial activation and stress index (EASIX) (Deng et al., 2024) was computed as: EASIX = lactate dehydrogenase (U/L) × serum creatinine (mg/dL)/platelet count (10^9^/L). The triglyceride-glucose (TyG) index (Yang et al., 2023) was calculated via the equation TyG = ln [fasting triglycerides (mg/dL) × fasting blood glucose (BG) (mg/dL)/2]. The stress hyperglycemia ratio (SHR) (Li et al., 2024) was calculated using the formula: SHR = fasting BG (mg/dL)/(1.59 × HbA1c (%)-2.59). The prognostic nutritional index (PNI) (Li et al., 2026) is calculated as: PNI = 10 × serum albumin (g/dL) + 5 × lymphocyte count (10^9^/L).

### Outcome definitions

The primary outcome was the development of AKI during ECMO support. AKI is diagnosed in accordance with the 2012 KDIGO criterion (Kidney Disease: Improving Global Outcomes (KDIGO) Acute Kidney Injury Work Group, 2012) as one of the following: 1) Serum Cr increase ≥ 26.5 μmol/L within 48 hours; 2) Serum Cr increased to ≥ 1.5 times the baseline within 7 days; 3) UO < 0.5 mL/kg/h for 6 hours. Baseline serum Cr was determined as follows: 1) When serum Cr was assayed within 7 days before ICU admission, the measured or averaged value served as the baseline; 2) When no serum Cr result was available within this 7-day period, historical serum Cr records collected 7 to 365 days prior to ICU entry were extracted retrospectively, and their average was used for baseline calculation.

### Two-phased model development and validation

#### Missing data processing

Using stratified random sampling from the rsample package, 344 patients receiving ECMO support were allocated randomly to derivation (n = 238) and validation (n = 106) cohorts with a 7:3 grouping ratio. Little’s MCAR test was adopted to determine whether missing data satisfied the missing completely at random (MCAR) assumption. Multiple imputation with chained equations (MICE) was implemented to handle missing values. Multiple imputation was conducted after cohort splitting and only within the derivation cohort. Ten imputed datasets were generated; the prediction model was fitted separately on each dataset. Final patient-level risk estimates were derived by averaging predicted probabilities across the ten fitted models. The proportion of missing data for variables is provided in **Supplementary Material 1**.

#### Preliminary feature selection

Feature selection was separately conducted for the pre-ECMO and on-ECMO datasets following two sequential steps. First, univariate screening was conducted on the derivation cohort, retaining variables marginally linked to AKI outcomes (*P* < 0.10) to strike a balance between lowering type II error and initial model comprehensiveness. Second, the Boruta algorithm ranked features by importance metrics, reserving variables above the predefined thresholds to enhance model precision and lower dimensionality. The parameter settings are as follows: *P*-value threshold = 0.01, Iterations = 100, Bonferroni’s method = TRUE.

#### ML model development and validation

A modeling framework for ECMO-AKI was developed and evaluated in pre-ECMO and on-ECMO datasets. The methodology was as follows:

1. ML algorithm combinations: Twelve classical ML algorithms, including Stepglm, extreme gradient boosting (XGBoost), linear discriminant analysis (LDA), NaiveBayes, support vector machine (SVM), gradient boosting machine (GBM), plsRglm, least absolute shrinkage and selection operator (LASSO), random forest (RF), glmBoost, ridge regression, and Enet, were adopted. Grid-search hyperparameter tuning for each algorithm was performed exclusively on the derivation cohort. Through integrating 12 ML algorithms with different parameters, multiple distinct combinations were generated.
2. Model development: Among these, RF, LASSO, Stepglm, and glmBoost perform feature-selection functions within the derivation cohort. The analysis workflow consisted of feature screening followed by model training. For integrated model combinations, the former selects features, and the latter are applied for model construction.
3. Model evaluation: The distinct ML combinations were evaluated by AUC in the derivation cohort. A 1000-bootstrap resampling approach was adopted to yield bias-corrected AUC values. In the validation cohort, each model’s performance was calculated as an AUC estimate.
4. Model selection: The final predictive model for ECMO-AKI was selected based on bias-corrected AUC values from the derivation cohort, with additional considerations for model parsimony and generalization stability, as assessed by the AUC discrepancy between the derivation cohort and the held-out validation cohort.

### Model evaluation

Variance inflation factor (VIF) and Spearman correlation analysis were adopted to test multicollinearity among variables of the ECMO-AKI model. Multicollinearity was identified when VIF > 5, tolerance < 0.2, or pairwise Spearman correlation coefficient > 0.5.

The discrimination performance of the ECMO-AKI model was evaluated via the area under the curve (AUC) and corresponding 95% confidence interval (CI) from receiver operating characteristic (ROC) and precision-recall (PR) curves. For the derivation dataset, 1000-bootstrap resampling was performed to compute bias-corrected AUC-ROC. For the hold-out validation cohort, 1000-bootstrap resampling was used to derive 95% confidence intervals for AUC-ROC and AUC-PR. Additional evaluation metrics included sensitivity, specificity, positive predictive value (PPV), negative predictive value (NPV), precision, accuracy, F1-score, Matthews correlation coefficient (MCC), prevalence, recall, false negative rate (FNR), and false positive rate (FPR). Model calibration was visualized with a calibration curve to compare predicted probabilities against observed probabilities. Calibration was further quantified using the Brier score (optimal value = 0; values > 0.3 indicate unsatisfactory calibration) and calibration slope (optimal value = 1). Decision curve analysis (DCA) was implemented to assess clinical utility by calculating the net benefit across varying risk thresholds, comparing the ECMO-AKI model with the baseline.

Post-hoc Bootstrap resampling and 5-fold cross-validation were applied to evaluate the reliability of the ECMO-AKI prediction model across the full combined dataset. By partitioning the dataset into training and test subsets, cross-validation enables performance comparison among multiple models even with limited sample size. This approach avoids overreliance on a single data split and delivers a rigorous evaluation of model robustness.

Multivariable logistic regression identified the ECMO-AKI model as an independent risk predictor for AKI in patients undergoing ECMO, after covariate adjustment. Stratified subgroup analyses based on distinct clinical characteristics were performed to explore population-specific effect differences. The likelihood ratio test was adopted to assess interaction effects between the ECMO-AKI model and the stratified clinical variables.

### Sensitivity analysis

Considering that serum Cr are the diagnostic criteria for AKI, the Cr and EASIX (directly incorporating Cr) may artificially artificially enhance model performance when included as predictors. Accordingly, it is necessary to conduct a sensitivity analysis by excluding the above variables to further evaluate the model’s true predictive power.

Death prior to AKI onset constitutes a competing event that may lead to informative misclassification of patients who would otherwise have developed AKI. To address this concern, we performed the following pre-specified analyses. A sensitivity analysis was conducted by excluding all patients who died within 48 hours of ECMO initiation; both the pre-ECMOAKI and on-ECMOAKI models were re-evaluated in the remaining cohort.

### Model interpretability and web-based prediction calculator

Shapley Additive Explanations (SHAP) analysis (He et al., 2025) was implemented to interpret the constructed ECMO-AKI prediction model. SHAP values were calculated to rank the relative importance of individual clinical predictors incorporated into the model. The SHAP framework delivers both global and local interpretability. Local interpretation enables individualized AKI risk prediction for single ECMO patients by entering patient-specific data into the model. For global interpretation, consistent quantitative attribution values are assigned to each feature, quantifying the association between input variables and predicted ECMO-AKI risk. Furthermore, SHAP dependence plots were adopted to visualize relationships and interaction effects between the ECMO-AKI and predictors.

For flexible predictive applications, the ECMO-AKI model was deployed in an open-access interactive Shiny web app. The online calculator permits convenient input of clinical indicators and automatic generation of ECMO-AKI risk estimates.

### Model comparison

Multiple ROC analyses were performed to compare the discriminative ability of the ECMO-AKI with the EASIX, Cr, APACHE II, and SOFA-2. Bias-corrected AUCs were computed using 1000 bootstrap replicates, and pairwise intergroup comparisons of AUCs were conducted with the DeLong test. Furthermore, DCA was used to quantify the net clinical benefit of the ECMO-AKI model relative to the EASIX, Cr, APACHE II, and SOFA-2.

### Two-phased model collaborative prediction framework

The concordance framework integrated predictions from the pre-ECMOAKI and on-ECMOAKI models. Optimal single cut-off values were identified in the derivation cohort via the Youden index and directly applied to the validation cohort without modification. Using these fixed thresholds, the concordance-based strategy classified patients into four categories: consensus high risk, downgraded risk (high to low), upgraded risk (low to high), and consensus low risk.

### Model features analysis

To further investigate the practical value of each feature incorporated into the ECMO-AKI model, we systematically analyzed the associations between the predicted ECMO-AKI risk and individual predictor variables. First, restricted cubic spline (RCS) regression combined with chi-square tests was adopted to assess potential nonlinear relationships between ECMO-AKI and the predictors. Second, threshold effect analysis was further performed to identify the optimal cutoff points of feature variables. Third, paired tests were adopted to compare the changing trends of predictive variables between the pre-ECMO and on-ECMO periods. Fourth, the latent class growth mixture model (LCGMM) was applied to explore the associations between longitudinal feature trajectories and the occurrence of ECMO-AKI. Predictive features were further collected at 24 hours after ECMO initiation. Analytical steps are outlined below. (1) Latent class growth models (LCGM) with 2 to 4 latent classes were tested, and the optimal class number was chosen according to the Bayesian Information Criterion (BIC) and clinical interpretability. (2) Growth mixture models (GMM) incorporating two random-effect structures (random intercept and random slope) were fitted to longitudinal trajectories to pick the optimal model. Subjects were allocated to trajectory subgroups by their maximum posterior probability; an average posterior probability > 0.7 guaranteed robust classification. Each latent class was constrained to include ≥ 10% of the total cohort to avoid overfitting and maintain model parsimony.

### Statistical analysis

R software (R version 4.3.1), SPSS Statistics 22.0, GraphPad Prism 8.0, and Python-based DecisionLinnc 1.0 were utilized to perform the statistical analysis. Normally distributed continuous variables were presented as mean ± standard deviation (SD). Intergroup comparisons were conducted using the independent samples t-test, while paired t-tests were applied for paired data comparisons. Skewed continuous variables were reported as median (interquartile range, IQR; P25-P75). Between-group comparisons were performed with the Mann-Whitney U test, whereas paired comparisons were conducted using the Wilcoxon signed-rank test. Categorical variables were summarized as counts (percentages), with intergroup comparisons performed using the chi-square test or Fisher’s exact test when applicable. Two-tailed *P* < 0.05 was defined as statistically significant unless otherwise specified.

## Results

### Study population and patient characteristics

In total, 401 ECMO-supported patients were screened; 57 were excluded for predefined reasons, leaving 344 patients in the final analytical cohort. Exclusions included pre-existing stage 5 CKD or established AKI (n=14), prior RRT (n=11), overall missing data > 15% (n=2), and death within 24 hours of ECMO initiation (n=30). The final analytical cohort included 344 patients, among whom 200 developed AKI during ECMO support, corresponding to an incidence rate of 58.14%. Time from ECMO cannulation to AKI ascertainment among 200 AKI patients: minimum 10.5 h, median 67.2 h, maximum 300 h. Comparisons of baseline demographics, clinical characteristics, laboratory findings, ECMO-related features, fluid management, severity scores, interventions, and clinical outcomes were performed between the AKI and no AKI groups separately for the pre-ECMO and on-ECMO datasets (**Tables 1**, **2**; **Supplementary Material 2**), which demonstrated substantial disparities between the two groups. It is worth noting that more variables differed significantly between the two groups in the on-ECMO dataset than in the pre-ECMO dataset. In terms of medication management and clinical outcomes, compared with the no AKI group, it was observed that vasoactive agent administration, MVT, ECMOT, hospital/ICU LOS, and ICU mortality were significantly elevated in the AKI group.

**Table 1:** Baseline characteristics and biochemical indicators between AKI and non-AKI groups in the pre-ECMO dataset.

Variable	Overall	No AKI	AKI	*P*-value
	n = 344	n = 144	n = 200	
Age, y	49.55 (16.92)	47.92 (16.06)	50.72 (17.46)	0.125
Gender, Female	99 (28.78%)	53 (36.81%)	46 (23.00%)	0.005
CPRT, min	6.75 (0.00 - 51.00)	1.65 (0.00 - 35.25)	11.75 (0.00 - 59.50)	0.001
PH	7.27 (0.20)	7.30 (0.18)	7.25 (0.21)	0.029
PaCO_2_, mmHg	46.25 (21.69)	45.86 (20.16)	46.53 (22.77)	0.774
PaO_2_, mmHg	77.75 (54.50 - 168.50)	82.50 (58.50 - 177.30)	73.00 (49.50 - 162.00)	0.943
Lactate, mmol/L	7.80 (2.50 - 15.00)	4.55 (1.66 - 10.53)	9.60 (3.43 - 15.00)	<0.001
HCO3-, mmol/L	21.31 (7.43)	21.65 (6.31)	21.07 (8.15)	0.455
PaO_2_/FiO_2_	136.00 (79.49 - 256.50)	148.13 (93.70 - 256.70)	127.61 (66.50 - 256.50)	0.822
HR, beat	100.08 (33.68)	101.15 (28.85)	99.31 (36.82)	0.605
RR, times	17.87 (7.36)	17.70 (6.18)	18.00 (8.12)	0.704
SBP, mmHg	94.88 (28.72)	100.40 (28.18)	90.91 (28.52)	0.002
DBP, mmHg	64.90 (16.18)	68.05 (16.18)	62.63 (15.83)	0.002
MAP, mmHg	74.81 (18.02)	78.72 (17.51)	72.00 (17.91)	<0.001
WBC, ×10^9^/L	14.04 (9.71 - 20.02)	13.37 (9.71 - 18.58)	14.85 (9.64 - 21.12)	0.215
Neutrophils, ×10^9^/L	11.38 (7.37 - 16.67)	11.25 (7.90 - 14.95)	11.58 (7.27 - 17.41)	0.563
Lymphocytes, ×10^9^/L	1.52 (0.96 - 2.56)	1.56 (1.00 - 2.38)	1.48 (0.87 - 2.66)	0.743
Hemoglobin, g/L	109.73 (30.28)	112.11 (27.30)	108.01 (32.22)	0.204
PLT, ×10^9^/L	161.50 (101.00 - 231.00)	193.00 (127.00 - 254.50)	139.00 (69.50 - 213.50)	<0.001
RBC, ×10^9^/L	3.45 (0.96)	3.55 (0.89)	3.38 (0.99)	0.091
HCT	0.31 (0.08)	0.32 (0.07)	0.31 (0.09)	0.215
RDW, %	15.47 (3.52)	14.95 (2.86)	15.85 (3.88)	0.015
CRP, ng/L	34.24 (8.25 - 84.00)	35.24 (8.12 - 72.68)	33.47 (8.54 - 90.73)	0.416
ProCT, ng/mL	4.50 (0.67 - 20.95)	2.55 (0.33 - 14.02)	5.83 (1.29 - 27.40)	0.055
Potassium, mmol/L	4.13 (0.92)	4.08 (0.83)	4.16 (0.98)	0.38
Sodium, mmol/L	142.40 (6.89)	141.14 (6.22)	143.30 (7.21)	0.003
Calcium, mmol/L	1.02 (0.22)	1.04 (0.18)	1.01 (0.24)	0.171
Cr, μmol/L	128.65 (89.85 - 194.15)	109.85 (73.95 - 155.35)	152.90 (103.00 - 216.10)	<0.001
BUN, mg/dL	9.12 (7.11 - 11.43)	8.72 (6.84 - 10.44)	9.51 (7.59 - 12.01)	0.012
GFR, ml/(min·1.73 m²)	59.22 (40.78 - 82.24)	68.83 (45.35 - 99.76)	53.54 (35.53 - 74.91)	<0.001
ALT, U/L	110.00 (40.50 - 340.00)	67.00 (32.50 - 223.00)	144.50 (53.00 - 469.00)	0.005
AST, U/L	298.50 (69.00 - 981.50)	183.00 (53.00 - 738.50)	445.50 (104.50 - 1, 422.50)	0.001
LDH, U/L	830.50 (437.50 - 1, 791.00)	637.00 (414.25 - 1, 198.31)	1, 101.00 (469.00 - 2, 399.45)	<0.001
TP g/L	51.30 (10.33)	53.89 (9.86)	49.43 (10.29)	<0.001
Albumin, g/L	32.06 (7.54)	32.31 (6.78)	31.88 (8.06)	0.593
TBI, μmol/L	15.66 (10.35 - 27.69)	14.42 (10.30 - 21.88)	17.95 (10.37 - 30.17)	0.008
DBI, μmol/L	7.41 (4.37 - 14.93)	6.83 (4.01 - 10.62)	8.25 (4.73 - 17.30)	0.015
IBI, μmol/L	7.66 (5.04 - 12.93)	7.61 (5.04 - 11.15)	7.71 (5.07 - 13.98)	0.074
ProBNP, pg/mL	3, 406.30 (768.37 - 8, 122.72)	2, 401.78 (321.00 - 8, 186.10)	4, 095.00 (1, 430.00 - 7, 946.06)	0.107
CK, U/L	1, 956.55 (420.55 - 5, 862.08)	1, 448.00 (378.55 - 4, 283.50)	3, 000.00 (493.30 - 6, 667.40)	0.003
CK-MB, U/L	124.64 (33.75 - 409.58)	70.50 (25.90 - 291.72)	234.25 (38.92 - 516.78)	0.002
BG, mmol/L	9.39 (4.06)	9.49 (4.02)	9.33 (4.11)	0.713
HbA1c, %	6.55 (1.21)	6.57 (1.50)	6.53 (0.96)	0.769
PT, s	15.90 (13.15 - 20.70)	14.40 (12.15 - 17.65)	17.65 (14.40 - 22.90)	0.007
APTT, s	43.70 (30.70 - 77.05)	33.45 (27.75 - 66.20)	48.40 (34.75 - 86.70)	0.003
TT, s	22.70 (17.55 - 62.75)	21.35 (17.15 - 41.55)	24.35 (18.25 - 90.00)	0.082
FIB, g/L	2.55 (1.51 - 3.84)	2.86 (1.82 - 3.83)	2.25 (1.32 - 3.84)	0.983
EASIX	9.37 (2.78 - 24.20)	4.59 (1.94 - 11.63)	16.37 (4.93 - 39.13)	0.001
SHR	1.22 (0.48)	1.24 (0.50)	1.21 (0.47)	0.543
PNI	42.02 (11.75)	42.10 (10.58)	41.96 (12.54)	0.912

AKI, acute kidney injury; ECMO, extracorporeal membrane oxygenation; CPRT, cardiopulmonary resuscitation time; HR: heart rate; SBP, systolic blood pressure; DBP, diastolic blood pressure; MAP, mean arterial pressure; RR, respiratory rate; WBC, white blood cell; RBC, red blood cell; WBC, white blood cell; PLT, platelet; RDW, red blood cell volume distribution width; HCT,hematocrit; Cr, creatinine; CRP, C-reactive protein; ProCT, procalcitonin; BUN, blood urea nitrogen; GFR, glomerular filtration rate; AST, aspartate aminotransferase; ALT, alanineaminotransferase; LDH, lactate dehydrogenase; TP, total protein; TBI, total bilirubin; DBI, direct bilirubin; IBI, indirect bilirubin; PT, prothrombin time; APTT, activation partial thromboplastintime; TT, thrombin time; FIB, fibrinogen; ProBNP, pro-brain natriuretic peptide; CK, creatine kinase; CK-MB, creatine kinase isoenzymes; BG, blood glucose; HbA1c, glycated hemoglobin A1c;PNI, prognostic nutritional index; EASIX, endothelial activation and stress index; SHR, stress hyperglycemia ratio.

**Table 2:** Clinical characteristics and biochemical indicators between AKI and non-AKI groups in the on-ECMO dataset.

Variable	Overall	No AKI	AKI	*P*-value
	n = 344	n = 144	n = 200	
Catheterization time, min	28.35 (20.00 - 35.00)	27.75 (20.00 - 35.40)	29.00 (22.50 - 35.00)	0.391
ECMO				0.127
VA	265 (77.03%)	110 (76.39%)	155 (77.50%)	
VAV	5 (1.45%)	0 (0.00%)	5 (2.50%)	
VV	74 (21.51%)	34 (23.61%)	40 (20.00%)	
PH	7.40 (0.09)	7.41 (0.09)	7.39 (0.09)	0.054
PaCO_2_, mmHg	38.25 (10.41)	38.72 (6.94)	37.91 (12.33)	0.438
PaO_2_, mmHg	140.10 (92.00 - 191.50)	136.65 (95.40 - 181.70)	140.90 (91.10 - 197.00)	0.076
Lactate, mmol/L	2.10 (1.00 - 8.75)	1.40 (0.90 - 3.46)	2.65 (1.20 - 14.25)	<0.001
HCO3-, mmol/L	22.41 (6.03)	23.42 (5.69)	21.68 (6.18)	0.007
PaO_2_/FiO_2_	252.90 (184.65 - 358.00)	260.15 (195.98 - 358.00)	250.25 (168.53 - 355.50)	0.702
HR, beat	88.68 (25.16)	87.86 (21.31)	89.27 (27.64)	0.594
RR, times	15.67 (4.34)	15.05 (4.05)	16.12 (4.50)	0.021
SBP, mmHg	100.22 (25.29)	106.29 (23.52)	95.85 (25.68)	<0.001
DBP, mmHg	61.76 (11.98)	63.69 (11.81)	60.38 (11.94)	0.011
MAP, mmHg	73.72 (15.31)	77.62 (15.31)	70.91 (14.71)	<0.001
WBC, ×10^9^/L	13.69 (10.15 - 19.02)	12.81 (10.10 - 16.46)	14.17 (10.27 - 20.64)	0.045
Neutrophils, ×10^9^/L	10.30 (7.00 - 15.36)	10.19 (7.00 - 13.29)	10.41 (7.00 - 16.64)	0.231
Lymphocytes, ×10^9^/L	1.38 (0.78 - 2.23)	1.53 (0.89 - 2.36)	1.19 (0.73 - 2.07)	0.006
Hemoglobin, g/L	104.80 (31.59)	111.42 (29.41)	100.03 (32.31)	<0.001
PLT, ×10^9^/L	140.50 (56.00 - 216.50)	179.00 (105.00 - 254.50)	111.00 (40.00 - 181.50)	<0.001
HCT	0.32 (0.10)	0.34 (0.09)	0.30 (0.10)	<0.001
RDW, %	14.24 (1.77)	14.15 (1.60)	14.30 (1.89)	0.436
CRP, ng/L	58.91 (12.20 - 111.53)	47.34 (10.06 - 105.17)	68.91 (13.46 - 123.81)	0.097
ProCT, ng/mL	4.04 (0.67 - 19.51)	1.49 (0.34 - 10.47)	5.76 (1.48 - 27.90)	0.049
Potassium, mmol/L	4.27 (1.17)	4.16 (1.11)	4.34 (1.21)	0.147
Sodium, mmol/L	143.40 (7.45)	142.41 (7.68)	144.11 (7.22)	0.039
Calcium, mmol/L	1.38 (0.31)	1.41 (0.30)	1.36 (0.31)	0.093
Cr, μmol/L	137.00 (91.95 - 190.20)	106.10 (77.60 - 162.93)	152.58 (107.84 - 207.20)	<0.001
BUN, mg/dL	11.15 (7.82 - 14.70)	9.85 (6.86 - 14.23)	11.73 (8.54 - 14.97)	0.094
GFR, ml/(min·1.73 m²)	58.50 (37.26 - 86.26)	63.84 (41.83 - 98.20)	53.17 (35.44 - 76.57)	0.005
ALT, U/L	82.00 (34.50 - 289.50)	56.00 (30.00 - 142.00)	116.00 (43.00 - 449.50)	0.575
AST, U/L	180.50 (60.00 - 798.00)	126.50 (46.50 - 382.00)	225.50 (84.00 - 951.50)	0.021
LDH, U/L	736.00 (481.20 - 1, 743.35)	608.00 (439.50 - 994.40)	946.50 (539.50 - 2, 588.50)	0.004
Triglyceride, mmol/L	1.43 (1.07 - 2.12)	1.30 (0.93 - 1.81)	1.56 (1.20 - 2.20)	0.003
TP g/L	54.07 (12.06)	56.98 (11.91)	51.97 (11.77)	<0.001
Albumin, g/L	32.35 (7.68)	33.58 (7.22)	31.46 (7.89)	0.01
TBI, μmol/L	21.24 (12.34 - 46.80)	16.57 (10.61 - 28.40)	27.81 (15.11 - 78.24)	<0.001
DBI, μmol/L	10.20 (5.40 - 26.60)	7.30 (4.22 - 12.82)	14.10 (6.69 - 51.55)	<0.001
IBI, μmol/L	10.29 (6.30 - 18.15)	9.36 (5.80 - 15.14)	12.27 (7.10 - 23.99)	<0.001
ProBNP, pg/mL	4, 619.02 (921.00 - 9, 906.12)	2, 913.40 (628.00 - 8, 210.00)	5, 450.00 (1, 368.56 - 11, 450.00)	0.036
CK, U/L	1, 777.50 (291.71 - 3, 749.10)	1, 369.39 (278.00 - 2, 788.85)	2, 154.85 (325.50 - 4, 958.40)	0.005
CK-MB, U/L	63.97 (34.45 - 170.50)	57.12 (28.20 - 107.62)	78.40 (36.15 - 248.50)	0.009
BG, mmol/L	10.61 (4.46)	9.86 (3.90)	11.16 (4.75)	0.006
PT, s	15.10 (12.55 - 19.65)	13.45 (12.00 - 17.50)	16.20 (13.20 - 20.55)	0.025
APTT, s	42.25 (28.90 - 68.75)	36.65 (27.85 - 66.20)	47.00 (30.50 - 72.75)	0.089
TT, s	22.05 (17.50 - 38.65)	21.55 (17.25 - 51.60)	22.20 (17.80 - 37.90)	0.329
FIB, g/L	2.94 (1.71 - 4.54)	3.10 (2.31 - 4.64)	2.72 (1.51 - 4.32)	0.621
EASIX	10.70 (3.42 - 41.11)	4.75 (2.30 - 11.62)	19.49 (7.24 - 88.38)	0.013
SHR	1.39 (0.60)	1.30 (0.52)	1.46 (0.65)	0.01
TyG	9.39 (0.69)	9.20 (0.69)	9.53 (0.66)	<0.001
PNI	40.52 (10.39)	42.79 (10.00)	38.88 (10.39)	<0.001
Input volume, mL	3, 374.95 (2, 616.50 - 4, 361.69)	3, 055.65 (2, 286.85 - 3, 987.84)	3, 648.35 (2, 856.00 - 4, 721.00)	<0.001
Output volume, mL	3, 024.80 (2, 101.90 - 3, 906.25)	3, 060.05 (2, 050.00 - 3, 782.50)	3, 015.00 (2, 134.40 - 4, 013.50)	0.288
Fluid balance, mL	168.50 (-555.65 - 1, 393.50)	105.85 (-500.75 - 572.95)	227.96 (-576.00 - 2, 208.57)	<0.001
24h UO, mL	1, 230.75 (232.50 - 1, 902.50)	1, 659.00 (920.00 - 2, 625.00)	806.00 (32.50 - 1, 456.75)	<0.001
SOFA-2	9 (6 - 13)	8 (5 - 9)	12 (8 - 14)	<0.001
APACHE II	24.45 (9.58)	20.09 (8.26)	27.59 (9.24)	<0.001
Hospital LOS, d	16.46 (7.04 - 30.92)	18.08 (9.72 - 30.92)	14.29 (5.47 - 30.83)	0.046
ICU LOS, d	9.75 (4.62 - 18.72)	8.81 (5.15 - 15.97)	9.95 (4.36 - 21.45)	0.033
MVT, d	7.18 (3.20 - 15.98)	5.69 (2.24 - 10.71)	8.99 (4.00 - 18.61)	0.001
ECMOT, d	4.97 (2.82 - 9.51)	4.03 (2.47 - 8.15)	5.85 (3.00 - 10.66)	0.028
Status				<0.001
Survival	133 (38.66%)	87 (60.42%)	46 (23.00%)	
Death	211 (61.34%)	57 (39.58%)	154 (77.00%)	

AKI, acute kidney injury; ECMO, extracorporeal membrane oxygenation; VA, venoarterial; VV, venovenous; VAV, venoarterial-venous; HR, heart rate; SBP, systolic blood pressure; DBP, diastolic blood pressure; MAP, mean arterial pressure; RR, respiratory rate; WBC, white blood cell; WBC, white blood cell; PLT, platelet; RDW, red blood cell volume distribution width; HCT,hematocrit; Cr, creatinine; CRP, C-reactive protein; ProCT, procalcitonin; BUN, blood urea nitrogen; GFR, glomerular filtration rate; AST, aspartate aminotransferase; ALT, alanineaminotransferase; LDH, lactate dehydrogenase; TP, total protein; TBI, total bilirubin; DBI, direct bilirubin; IBI, indirect bilirubin; PT, prothrombin time; APTT, activation partial thromboplastintime; TT, thrombin time; FIB, fibrinogen; ProBNP, pro-brain natriuretic peptide; CK, creatine kinase; CK-MB, creatine kinase isoenzymes; BG, blood glucose; HbA1c, glycated hemoglobin A1c;PNI, prognostic nutritional index; EASIX, endothelial activation and stress index; SHR, stress hyperglycemia ratio; TyG, triglyceride-glucose index; UO, urinary output; SOFA-2, sequential organ failure assessment 2; APACHE II, acute physiology and chronic health evaluation II; LOS, length of stay; ICU, intensive care unit; LOS, length of stay; MVT, mechanical ventilation time; ECMOT, extracorporeal membrane oxygenation time.

### Preliminary feature selection

The derivation (n = 238) and validation (n = 106) cohorts exhibited balanced baseline characteristics across pre-ECMO and on-ECMO datasets, with *P* > 0.05 and standardized mean differences (SMD) < 0.20 for all features (**Supplementary Materials 3, 4**). Univariate difference analysis screened out 28 and 39 candidate features significantly correlated with ECMO-AKI in the pre-ECMO and on-ECMO datasets, respectively (*P* < 0.10). Subsequently, the Boruta algorithm identified 12 features (EASIX, Cr, cardiopulmonary resuscitation time (CPRT), activation partial thromboplastin time (APTT), gender, prothrombin time (PT), procalcitonin (ProCT), lactate, direct bilirubin (DBI), creatine kinase isoenzymes (CK-MB), total bilirubin (TBI), total protein (TP)) from the pre-ECMO dataset (**Figure 2A**) and 16 features (EASIX, TBI, DBI, TyG, ProCT, Cr, platelet (PLT), systolic blood pressure (SBP), triglyceride, lactate, PNI, mean arterial pressure (MAP), lymphocytes, white blood cell (WBC), PT, hematocrit (HCT)) from the on-ECMO dataset (**Figure 2B**), according to predictive importance ranking (**Supplementary Material 5**).

**Figure 2:**
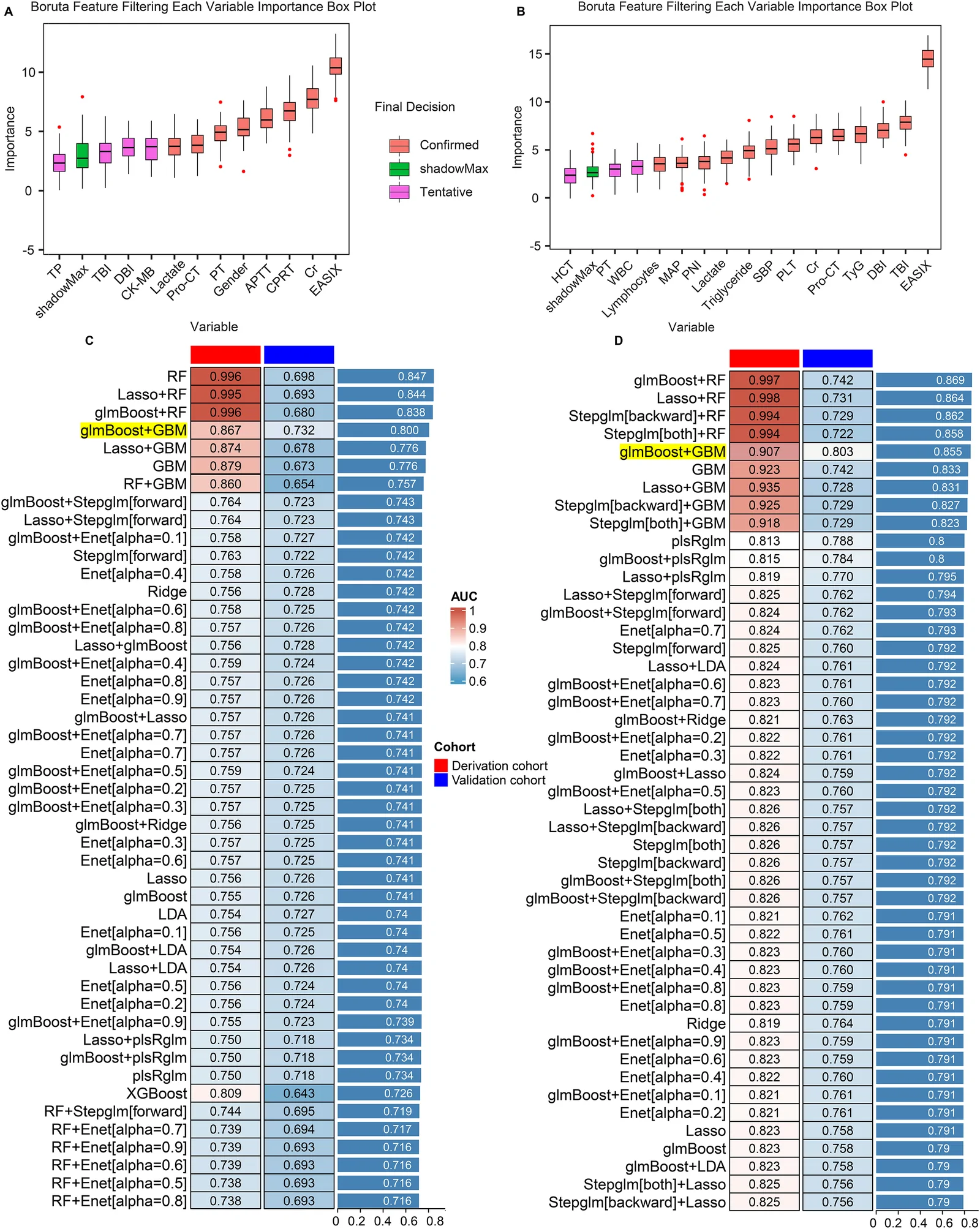
Feature selection and two-phased machine learning (ML) model development and validation. **(A, B)** Important characteristic variables identified by the Boruta algorithm in pre-ECMO **(A)** and on-ECMO **(B)** datasets. The horizontal axis shows the names of variables, and the vertical axis shows the Z-score of variables. The boxplot shows the Z-score of variables during the model calculation process. **(C, D)** An innovative modeling framework using 12 classical ML-based integrative procedure and further calculated the area under curve (AUC) of each model in derivation and validation cohorts for pre-ECMOAKI **(C)** and on-ECMOAKI **(D)** models. AKI, acute kidney injury; ECMO, extracorporeal membrane oxygenation.

Notably, in the on-ECMO dataset, following Boruta screening, triglycerides and lymphocytes were excluded due to high collinearity (r > 0.6) with TyG and PNI; the composite indices TyG and PNI with higher Boruta importance were kept. Although rejected by Boruta, SHR was preserved given its clinical and pathophysiological relevance to ECMO-AKI (Li et al., 2024). A total of 15 variables entered the on-ECMOAKI model-building candidate pool.

### Two-phased ML model development and validation

For the development of a real-time, dependable diagnostic model of ECMO-AKI, a staged prediction structure was adopted, with two independent models established separately from the pre-ECMO and on-ECMO datasets.

For pre-ECMOAKI model, we implemented a modeling framework that combined 12 classic ML algorithms into 66 distinct algorithm combinations in the derivation cohort, with 12 features imputed and incorporated into the analytical pipeline. Among these ML, LASSO, glmBoost, RF, and Stepglm conducted feature selection, selecting 10, 9, 7, and 5 features, respectively. The AUC for each algorithm was determined separately in the derivation and validation cohorts, alongside the overall mean AUC calculated across the two cohorts. Consequently, among these predictive models, “RF”, “Lasso+RF”, “glmBoost+RF”, and “glmBoost+GBM” demonstrated superior predictive performance (**Figure 2C**; **Supplementary Figure S1**). Nevertheless, “RF”, “Lasso+RF”, and “glmBoost+RF” achieved near-perfect discrimination (AUC ≥ 0.995) in derivation cohort but substantially degraded performance (AUC ≤ 0.698) in validation cohort, suggesting severe overfitting. Taken together, considering predictive discrimination, goodness-of-fit, and model simplicity, the “glmBoost+GBM” model (AUC = 0.867 in derivation cohort, AUC = 0.732 in validation cohort) incorporating nine variables (gender, CPRT, lactate, Cr, TP, TBI, ProCT, PT, and EASIX) was identified as the optimal predictive model for ECMO-AKI.

For the on-ECMOAKI model, the same model construction workflow was implemented. A total of 91 algorithm combinations were generated from 12 ML algorithms based on the 15 imputed features. LASSO, glmBoost, RF, and Stepglm were applied for feature selection, retaining 11, 10, 5, and 9 features, respectively. Similarly, the glmBoost+GBM model was determined as the optimal predictive model for ECMO-AKI, including ten predictors (lactate, MAP, WBC, EASIX, Cr, TBI, PT, SHR, PNI, TyG), with an AUC of 0.907 in derivation cohort, 0.803 in validation cohort, and average AUC of 0.855 (**Figure 2D**; **Supplementary Figure S2**).

For pre-ECMOAKI and on-ECMOAKI models, detailed data on model performance, feature selection results for each algorithm, individual patient risk scores, and predictive classification outcomes are provided in **Supplementary Materials 6, 7**. Detailed hyperparameter tuning information, including search ranges, optimization metric and final selected value for 12 ML, is provided in **Supplementary Material 8**.

### Two-phased ML model evaluation

First, VIF < 2, tolerance > 0.5, and inter-variable correlation coefficients < 0.5 were observed across pre-ECMOAKI and on-ECMOAKI models, demonstrating no obvious multicollinearity (**Supplementary Material 9**).

Second, ROC analysis revealed that the pre-ECMOAKI and on-ECMOAKI models displayed superior predictive performance, achieving a bootstrap-adjusted AUC of 0.867 (95%CI: 0.819-0.911) and 0.907 (95%CI: 0.870-0.941) in derivation cohort (**Figure 3A**), bootstrap-adjusted AUC of 0.732 (95%CI: 0.678-0.780) and 0.803 (95%CI: 0.711-0.889) in validation cohort (**Figure 3B**), bootstrap-adjusted AUC of 0.820 (95%CI: 0.768-0.861) and 0.877 (95%CI: 0.841-0.911) in overall cohort (**Figure 3C**), respectively. The AUC of PR curves reached 0.848 (95%CI: 0.807-0.889) in pre-ECMOAKI model (**Figure 3D**) and 0.905 (95%CI: 0.869-0.941) in on-ECMOAKI model (**Figure 3E**), respectively. For the pre-ECMOAKI model, the sensitivity, specificity, PPV, NPV, and F1-score were 0.795, 0.743, 0.811, 0.723, and 0.803, respectively. The corresponding metrics for the on-ECMOAKI model were 0.790, 0.826, 0.863, 0.739, and 0.825. The FNR, FPR, MCC, precision, accuracy, and recall were computed for the pre-ECMOAKI and on-ECMOAKI models (**Supplementary Material 10**).

**Figure 3:**
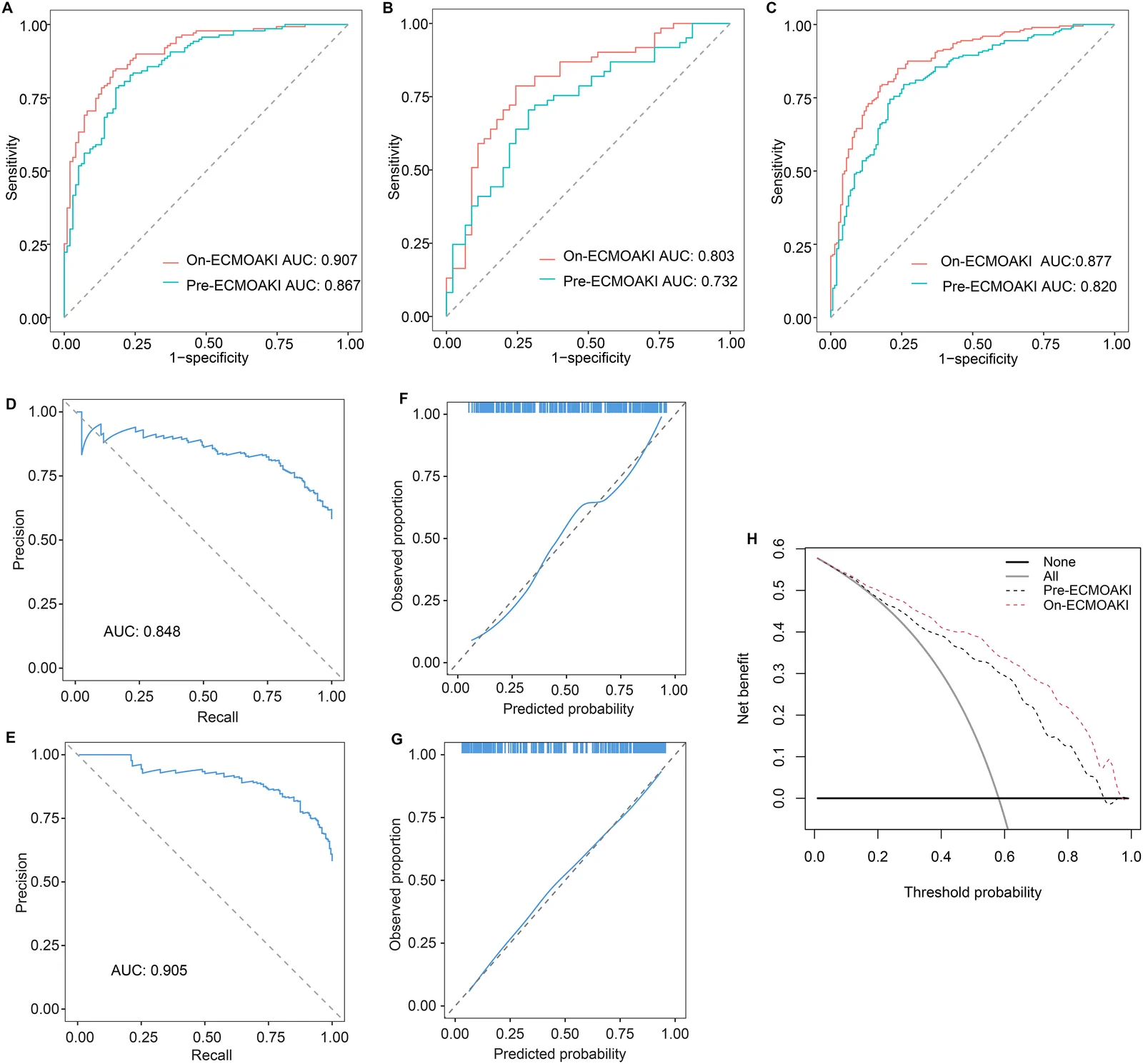
Comprehensive evaluation of predictive performance, calibration, and clinical utility of pre-ECMOAKI and on-ECMOAKI models in predicting ECMO-AKI. **(A–C)** Receiver operating characteristic (ROC) curves with bootstrap-adjusted AUC values to evaluate and comapre predictive performance of pre-ECMOAKI and on-ECMOAKI models in derivation **(A)**, validation **(B)** and overall **(C)** cohorts. **(D, E)** precision recall (PR) curves with AUC values to evaluate predictive efficacy of pre-ECMOAKI **(D)** and on-ECMOAKI **(E)** models. **(F, G)** Calibration curves for pre-ECMOAKI **(F)** and on-ECMOAKI **(G)** models. X-axis is predicted probability of ECMO-AKI. Y-axis is observed probability of ECMO-AKI. **(H)** Decision curve analysis (DCA) was applied to evaluate and compare the clinical usefulness of pre-ECMOAKI and on-ECMOAKI models. The Y-axis represents the net benefit. The black line represents the hypothesis that no patients treatment. The X-axis represents the threshold probability. The threshold probability is where the expected benefit of treatment is equal to the expected benefit of avoiding treatment.

Third, the calibration curves revealed excellent consistency between predicted and observed probabilities for both the pre-ECMOAKI and on-ECMOAKI models (**Figures 3F, G**). The pre-ECMOAKI and on-ECMOAKI models yielded Brier scores of 0.169 and 0.139, with calibration slopes of 1.004 and 1.000, respectively. DCA analysis showed that within the threshold probability ranges of 0.05-0.90 for the pre-ECMOAKI model and 0.03-0.95 for the on-ECMOAKI model, application of the established prediction models yielded higher net benefit than the “intervention all” or “non-intervention” strategies, confirming favorable clinical utility (**Figure 3H**).

Fourth, 5-fold cross-validation and bootstrap resampling were performed to further assess model reliability for the pre-ECMOAKI and on-ECMOAKI models. Consistently favorable performance was observed in ROC, PR, calibration curve, and DCA analyses across 5 distinct cohorts through 5-fold cross-validation (**Supplementary Figures S3**, **S4**) and bootstrap resampling (**Supplementary Figures S5**, **S6**), confirming robust predictive ability of both models. Moreover, the accuracy, prevalence, recall, F1-score, MCC, AUC, precision, specificity, FNR, and FPR were adopted to evaluate the pre-ECMOAKI and on-ECMOAKI models in 5 diverse cohorts (**Supplementary material 11**), which confirmed sustained stability for both prediction models.

Fifth, univariate logistic regression showed that the pre-ECMOAKI and on-ECMOAKI models were significantly correlated with elevated risk of ECMO-AKI in the crude unadjusted model, with OR = 2.11 (95%CI: 1.79-2.49) and OR = 2.33 (95%CI: 1.97-2.76), respectively (both *P* < 0.001; **Table 3**). Multivariable logistic regression analysis revealed that the pre-ECMOAKI and on-ECMOAKI models were independent predictors for ECMO-AKI risk following full covariate adjustment, with adjusted OR = 3.49 (95%CI: 2.35-5.21) and OR = 3.25 (95%CI: 2.23-4.75), respectively (both *P* < 0.001; **Table 3**).

**Table 3:** Univariable and multivariable logistic regression analysis for prediction of AKI in patients receiving ECMO support. .

Model	Pre-ECMOAKI		On-ECMOAKI	
	OR (95%CI)	*P*-value	OR (95% CI)	*P*-value
Coarse model	2.11 (1.79-2.49)	< 0.001	2.33 (1.97-2.76)	< 0.001
Model 1	2.12 (1.79-2.50)	< 0.001	2.32 (1.96-2.75)	< 0.001
Model 2	2.14 (1.80-2.56)	< 0.001	2.41 (1.99-2.92)	< 0.001
Model 3	3.49 (2.35-5.21)	< 0.001	3.25 (2.23-4.75)	< 0.001

Coarse model: no covariate was adjusted.

Model 1: Age and gender were adjusted.

Model 2: Age, gender, past medical history, ECMO indication, ECMO type, ECMO parameters, vasoactive drugs, albumin usage, catheterization time, and CPRT were adjusted.

Model 3: Age, gender, past medical history, ECMO indication, ECMO type, ECMO parameters, vasoactive drugs, albumin usage, catheterization time, CPRT, SOFA-2, APACHE II, PH, PaCO_2_, PaO_2_, HCO3-, PaO_2_/FiO_2_, HR, RR, SBP, DBP, MAP, WBC, neutrophils, lymphocytes, hemoglobin, PLT, RBC, HCT, RDW, CRP, ProCT, potassium, sodium, calcium, BUN, GFR, ALT, AST, LDH, albumin, DBI, IBI, ProBNP, CK, CK-MB, BG, HbA1c, APTT, TT, and FIB were adjusted.

AKI, acute kidney injury; ECMO, extracorporeal membrane oxygenation; CPRT, cardiopulmonary resuscitation time; HR, heart rate; SBP, systolic blood pressure; DBP, diastolic bloodpressure; MAP, mean arterial pressure; RR, respiratory rate; WBC, white blood cell; WBC, white blood cell; PLT, platelet; RDW, red blood cell volume distribution width; HCT, hematocrit; CRP, C-reactive protein; ProCT, procalcitonin; BUN, blood urea nitrogen;GFR, glomerular filtration rate; AST, aspartate aminotransferase; ALT, alanine aminotransferase; LDH, lactate dehydrogenase; TP, total protein; DB,: direct bilirubin; IBI, indirect bilirubin; APTT, activation partial thromboplastin time; TT, thrombin time; FIB, fibrinogen; ProBNP, pro-brain natriuretic peptide; CK, creatine kinase; CK-MB, creatine kinase isoenzymes; BG, blood glucose; HbA1c, glycated hemoglobin A1c; SOFA-2, sequential organ failure assessment 2; APACHE II, acute physiology and chronic health evaluation II.

Sixth, subgroup analyses were performed for the pre-ECMOAKI and on-ECMOAKI models to explore potential disparities across different populations. The results showed that both models remained significantly associated with higher ECMO-AKI risk across all subgroups (*P* < 0.001), apart from a few subgroups with limited sample sizes (**Table 4**; **Supplementary Material 12**). These findings verified that the two prediction models exhibited satisfactory overall robustness. Interaction analyses showed significant interactions of the pre-ECMOAKI model with output volume (*P* for interaction = 0.027) and catheterization time (*P* for interaction = 0.040). Meanwhile, the on-ECMOAKI model exhibited a significant interaction with catheterization time (*P* for interaction = 0.037).

**Table 4:** Subgroup analysis for pre-ECMOAKI model and on-ECMOAKI model in predicting AKI following ECMO.

		Pre-ECMOAKI model					On-ECMOAKI model				
Variable	Count	OR	Lower	Upper	*P* value	*P* for interaction	OR	Lower	Upper	*P* value	*P* for interaction
Overall	344	2.12	1.79	2.5	<0.001		2.32	1.96	2.75	<0.001	
Gender						0.309					0.281
Male	245										
Female	99										
Age, y						0.667					0.152
< 60	238	2.17	1.77	2.65	<0.001		2.66	2.11	3.36	<0.001	
≥ 60	106	2.1	1.51	2.93	<0.001		2.09	1.56	2.8	<0.001	
ECMO						0.801					0.902
VA	265	2.08	1.71	2.52	<0.001		2.33	1.92	2.83	<0.001	
VV	74	2.34	1.61	3.41	<0.001		2.66	1.73	4.07	<0.001	
VAV	5	1	0	Inf	1		1	0	Inf	1	
SOFA 2						0.813					0.458
≤ 9	194	2.01	1.61	2.51	<0.001		2.36	1.86	3	<0.001	
> 9	150	1.85	1.39	2.47	<0.001		2.02	1.56	2.61	<0.001	
APACHE II						0.514					0.549
≤ 15	58	2.38	1.47	3.86	<0.001		2.84	1.62	4.98	<0.001	
> 15	286	1.98	1.65	2.38	<0.001		2.23	1.86	2.68	<0.001	
Input volume, mL						0.16					0.588
≤ 3000	130	1.83	1.42	2.36	<0.001		2.21	1.69	2.88	<0.001	
> 3000	214	2.29	1.83	2.87	<0.001		2.39	1.91	2.99	<0.001	
Output volume, mL						0.027					0.241
≤ 3000	170	1.79	1.45	2.2	<0.001		2.13	1.71	2.65	<0.001	
> 3000	174	2.73	2.04	3.65	<0.001		2.64	2.01	3.47	<0.001	
Fulid balance, mL						0.123					0.763
≤ 0	146	2.6	1.91	3.55	<0.001		2.42	1.86	3.16	<0.001	
> 0	198	1.9	1.56	2.32	<0.001		2.29	1.83	2.86	<0.001	
24h UO, mL						0.232					0.06
≤ 1000	147	1.69	1.3	2.19	<0.001		1.81	1.42	2.3	<0.001	
> 1000	197	2.31	1.81	2.96	<0.001		2.65	2.04	3.44	<0.001	
Catheterization time, min						0.04					0.037
< 30	189	1.86	1.52	2.28	<0.001		2.04	1.66	2.5	<0.001	
≥ 30	155	2.75	2	3.79	<0.001		3.04	2.18	4.23	<0.001	
CPRT						0.887					0.338
No	145	2.11	1.64	2.72	<0.001		2.13	1.68	2.71	<0.001	
Yes	199	2.13	1.69	2.67	<0.001		2.69	2.06	3.51	<0.001	
Hospital LOS, d						0.709					0.192
≤ 14	155	2.18	1.68	2.82	<0.001		2.12	1.67	2.67	<0.001	
> 14	189	2.06	1.65	2.57	<0.001		2.62	2.03	3.38	<0.001	
ICU LOS, d						0.831					0.444
≤ 7	126	2.08	1.6	2.7	<0.001		2.21	1.69	2.87	<0.001	
> 7	218	2.16	1.73	2.7	<0.001		2.45	1.95	3.07	<0.001	
MVT, d						0.742					0.697
≤ 7	169	2.18	1.72	2.76	<0.001		2.25	1.78	2.83	<0.001	
> 7	175	2.06	1.61	2.63	<0.001		2.38	1.85	3.06	<0.001	
ECMOT, d						0.406					0.799
≤ 7	219	2.04	1.67	2.49	<0.001		2.37	1.91	2.94	<0.001	
> 7	125	2.33	1.71	3.19	<0.001		2.24	1.69	2.98	<0.001	

AKI, acute kidney injury; ECMO, extracorporeal membrane oxygenation; VA, venoarterial; VV, venovenous; VAV, venoarterial-venous; UO, urinary output; SOFA-2, sequential organ failure assessment 2; APACHE II, acute physiology and chronic health evaluation II; LOS, length of stay; ICU, intensive care unite; LOS, length of stay; MVT, mechanical ventilation time; ECMOT, extracorporeal membrane oxygenation time.

### Sensitivity analysis

Sensitivity analysis through excluding Cr and EASIX found that the pre-ECMOAKI and on-ECMOAKI models exhibited superior discrimination performance, with a bootstrap-adjusted AUC of 0.703 (95%CI: 0.644-0.758) and 0.784 (95%CI: 0.734-0.831), respectively. This suggests that the ECMO-AKI model retain satisfactory predictive capacity even after removing these two predictors, supporting their utility for risk prediction of ECMO-associated AKI.

Of the 211 in-hospital deaths, 38 (18.0%) occurred within 48 hours of ECMO initiation, including 24 of 200 (12.0%) in the AKI group and 14 of 144 (9.7%) in the non-AKI group; the 48-hour mortality did not differ significantly between groups (*P* = 0.53). After excluding the 38 patients who died within 48 hours, 306 patients remained. The discrimination of both models was essentially unchanged: the pre-ECMOAKI and on-ECMOAKI model retained a bootstrap-adjusted AUC of 0.816 (95%CI: 0.751-0.853) and 0.874 (95%CI: 0.837-0.908), respectively.

### SHAP-based model interpretation

For thorough interpretation of the included variables, SHAP analysis was implemented to assess the predictive contribution of each variable in the pre-ECMOAKI and on-ECMOAKI prediction models. **Figures 4A, B** display the top five predictive variables ranked by SHAP-based importance: EASIX, TP, lactate, TBI, and CPRT for the pre-ECMOAKI model; TyG, EASIX, PNI, TBI, and WBC for the on-ECMOAKI model.

**Figure 4:**
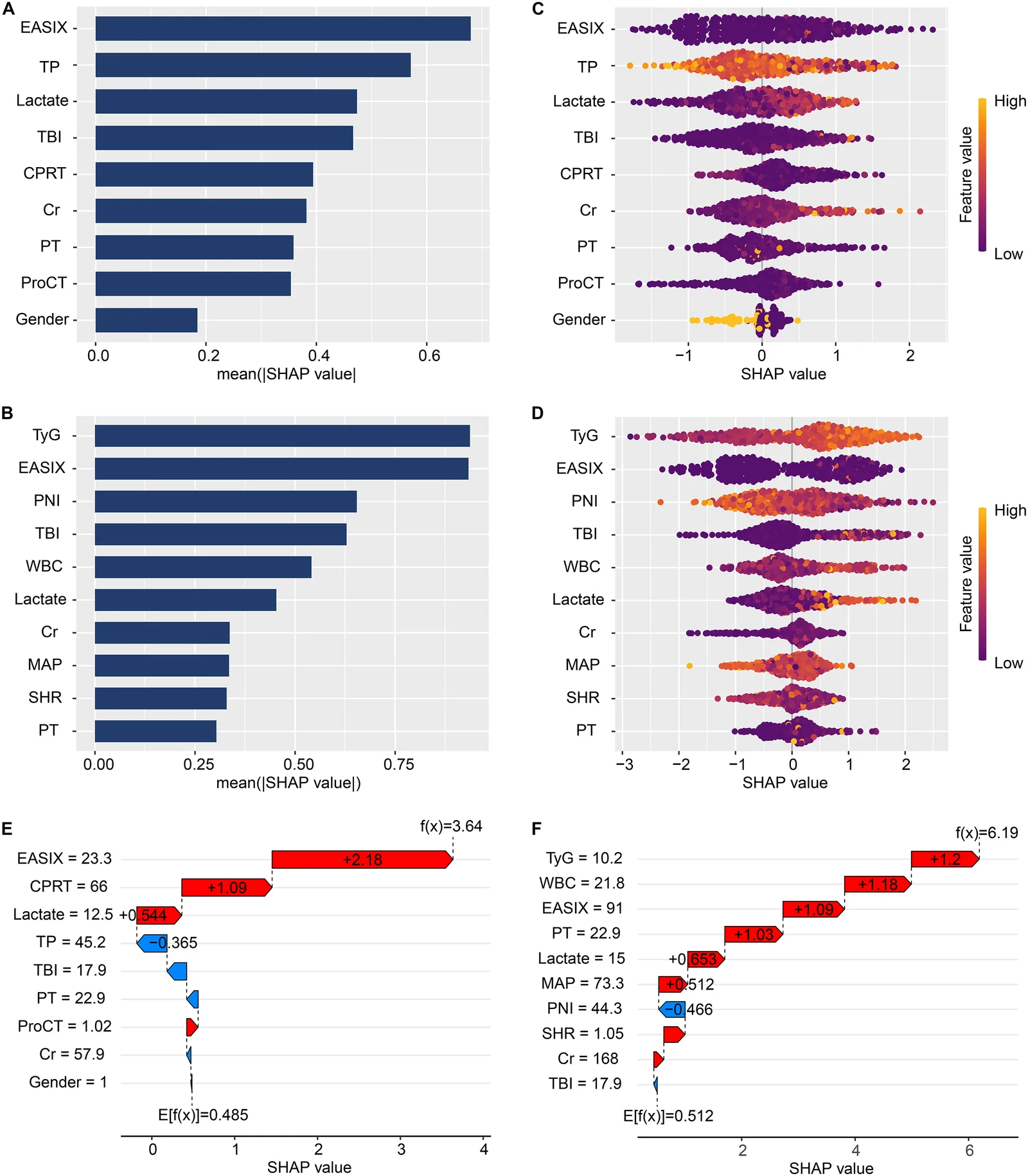
Global and local explanation by the Shapley Additive Explanations (SHAP) method. **(A, B)** Summary plot showed the features ranking by mean absolute SHAP values in pre-ECMOAKI **(A)** and on-ECMOAKI **(B)** models. **(C, D)** Global model explanation for pre-ECMOAKI **(C)** and on-ECMOAKI **(D)** models. Each variable name is shown on the left-hand side with the variable with the greatest contribution listed at the top. To the right of the variables, there are colored lines, which are individual points that correspond to observations in the population. A higher value for the variable is represented in yellow, while a lower value for the variable will be shown in purple. A value farther to the right (ie, a higher SHAP value) indicates that the variable is contributed to a prediction of a positive target, such as ECMO-AKI. **(E, F)** An example of risk factor analysis for a patient with ECMO-AKI which represented the individual ECMO towards the “AKI” class in pre-ECMOAKI **(E)** and on-ECMOAKI **(F)** models.

Global explanations: **Figures 4C, D** illustrate the distribution and value span of each predictor alongside their corresponding predictive effects in the pre-ECMOAKI and on-ECMOAKI models. EASIX, lactate, and TBI exhibited positive correlations with ECMO-AKI risk, while TP, PNI, and MAP showed negative correlations. Of note, variables may show opposing predictive directions for ECMO-AKI in distinct patients, while the effect magnitude of a given variable value does not change. The partial dependence plots visualized the global relationship between features and predicted ECMO-AKI risk probability (**Supplementary Figures S7**, **S8**).

Local explanations: To achieve individualized interpretation of ECMO-AKI risk, we dissected predictive variables and pinpointed risk contributors quantified by SHAP values. For the pre-ECMOAKI model, an ECMO-AKI patient with a total SHAP value of 3.64 developed high risk attributable to EASIX (23.3, SHAP = 2.18), CPRT (66 min, SHAP = 1.09) and lactate (12.5 mmol/L, SHAP = 0.544) (**Figure 4E**). Correspondingly, **Figure 4F** shows another ECMO-AKI individual from the on-ECMOAKI model with a total SHAP value reaching 6.19.

### Clinical implementation via web application

Given that predictive indicators are readily obtainable in clinical practice, we constructed web-based prediction tools to facilitate real-time clinical decision support. The calculators for the pre-ECMO AKI and on-ECMO AKI models can be accessed free of charge via the links below: https://lglcz.shinyapps.io/Pre-ECMO/ and https://lglcz.shinyapps.io/On-ECMO/.

### Comparison of two-stage ECMO-AKI model to APACHE II, SOFA-2, EASIX and Cr

Multiple ROC curve analyses showed that the pre-ECMOAKI and on-ECMOAKI models outperformed EASIX (AUC = 0.692 in pre-ECMO dataset; AUC = 0.735 in on-ECMO dataset), Cr (AUC = 0.667 in pre-ECMO dataset; AUC = 0.658 in on-ECMO dataset), APACHE II (AUC = 0.730) and SOFA-2 (AUC = 0.697) in predicting ECMO-AKI. Delong test confirmed that all inter-group differences in AUC were statistically significant (all *P* < 0.002), as presented in **Figures 5A, B**. Additionally, pairwise Delong comparisons indicated no significant AUC differences between EASIX, APACHE II, and SOFA-2. Finally, DCA curves revealed that both the pre-ECMOAKI and on-ECMOAKI models yielded superior clinical net benefit compared with the APACHE II, SOFA-2, EASIX and Cr (**Figures 5C, D**).

**Figure 5:**
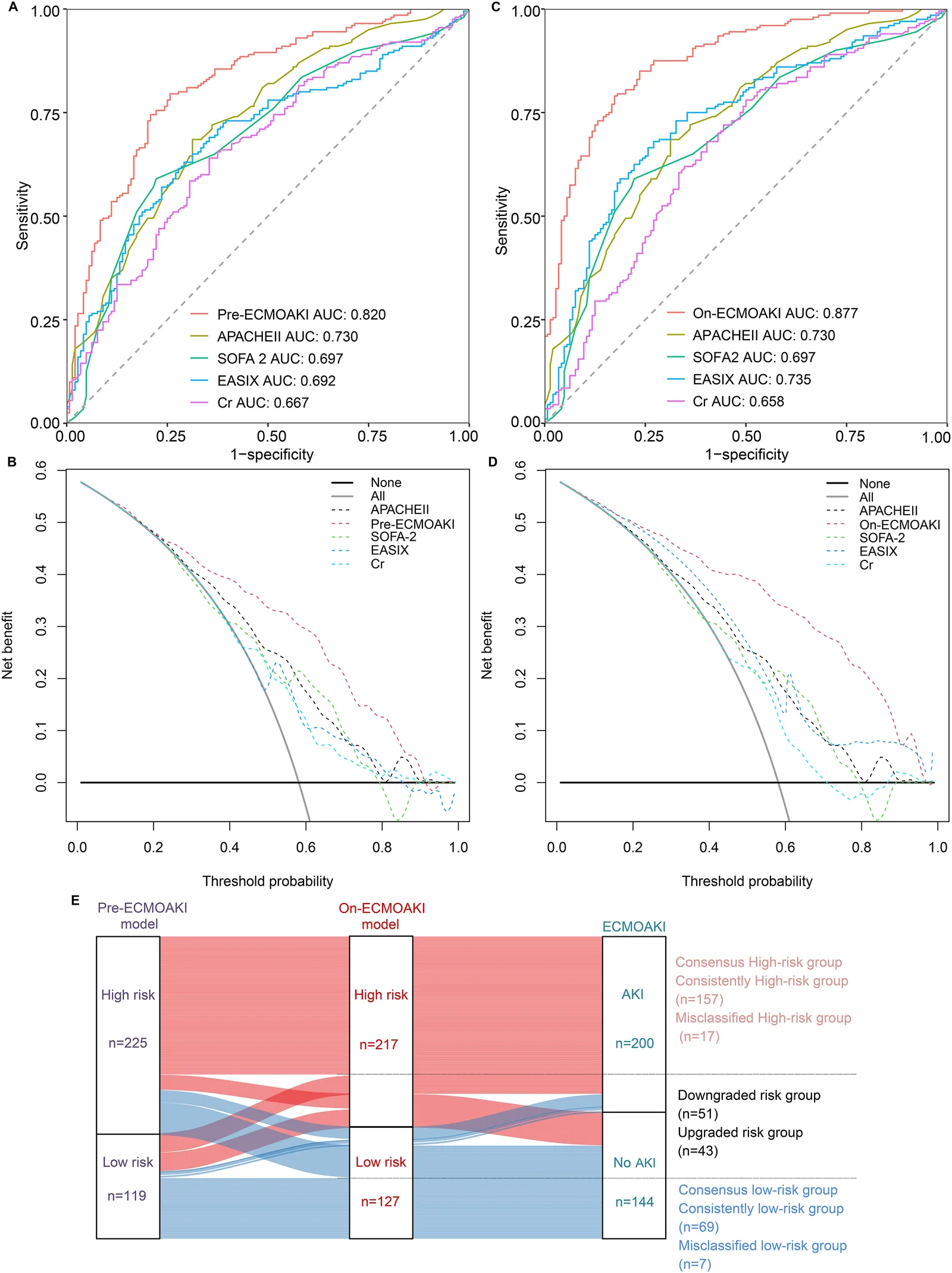
Comparison of two-stage ECMO-AKI model to other indicators, alongside synergistic prediction framework integrating the two models. **(A , B)** Multiple ROC analysis was performed to compare the diagnostic performance of the pre-ECMOAKI **(A)** and on-ECMOAKI **(B)** models against APACHE II, SOFA-2, EASIX and Cr. **(C, D)** DCA was applied to compare the clinical usefulness of pre-ECMOAKI **(C)** and on-ECMOAKI **(D)** models against APACHE II, SOFA-2, EASIX and Cr. **(E)** The Sankey plots illustrate the flow of patients across two time points based on the ECMO-AKI models’ risk predictions. Patients are stratified into four risk levels according to the integrated analysis of pre-ECMOAKI and on-ECMOAKI model predictions: consensus high-risk, consensus low-risk, upgraded risk and downgraded risk group. The light red flow represents patients in the consensus high-risk group who developed ECMO-AKI, while the light blue flow represents consensus high-risk patients who did not. The light blue flow indicates consensus low-risk patients who did not develop ECMO-AKI.

### Risk stratification based on the concordance prediction framework

To facilitate early and accurate risk stratification, we developed a combined risk assessment strategy incorporating the pre-ECMOAKI and on-ECMOAKI prediction models (**Figure 5E**). 225 patients were stratified into the high-risk group by the pre-ECMOAKI model; among them, 174 were still classified as high-risk based on the on-ECMOAKI model, and 157 patients eventually developed ECMO-AKI. In parallel, 119 patients were deemed low-risk per the pre-ECMOAKI model, and 76 retained low-risk status in the on-ECMOAKI model, among whom 69 did not develop ECMO-AKI. Within this synergistic prediction framework, the class-specific precision reached 90.23% (157/174) for ECMO-AKI events in consensus high-risk patients, and 90.79% (69/76) for non-ECMO-AKI events in consensus low-risk patients. The integrated risk stratification covered 72.67% of all enrolled subjects by defining these two consistent risk subgroups.

### Analysis of model predictors and ECMO-AKI risk

First, RCS regression analysis were applied to investigate nonlinear associations with optimised knot. Four predictors displayed nonlinear relationships with ECMO-AKI risk in pre-ECMOAKI model, as visualized in **Figures 6** and **Supplementary Figure 9**. Five predictors showed nonlinear associations with ECMO-AKI risk in on-ECMOAKI model, illustrated in **Figures 6** and **Supplementary Figure 10**.

**Figure 6:**
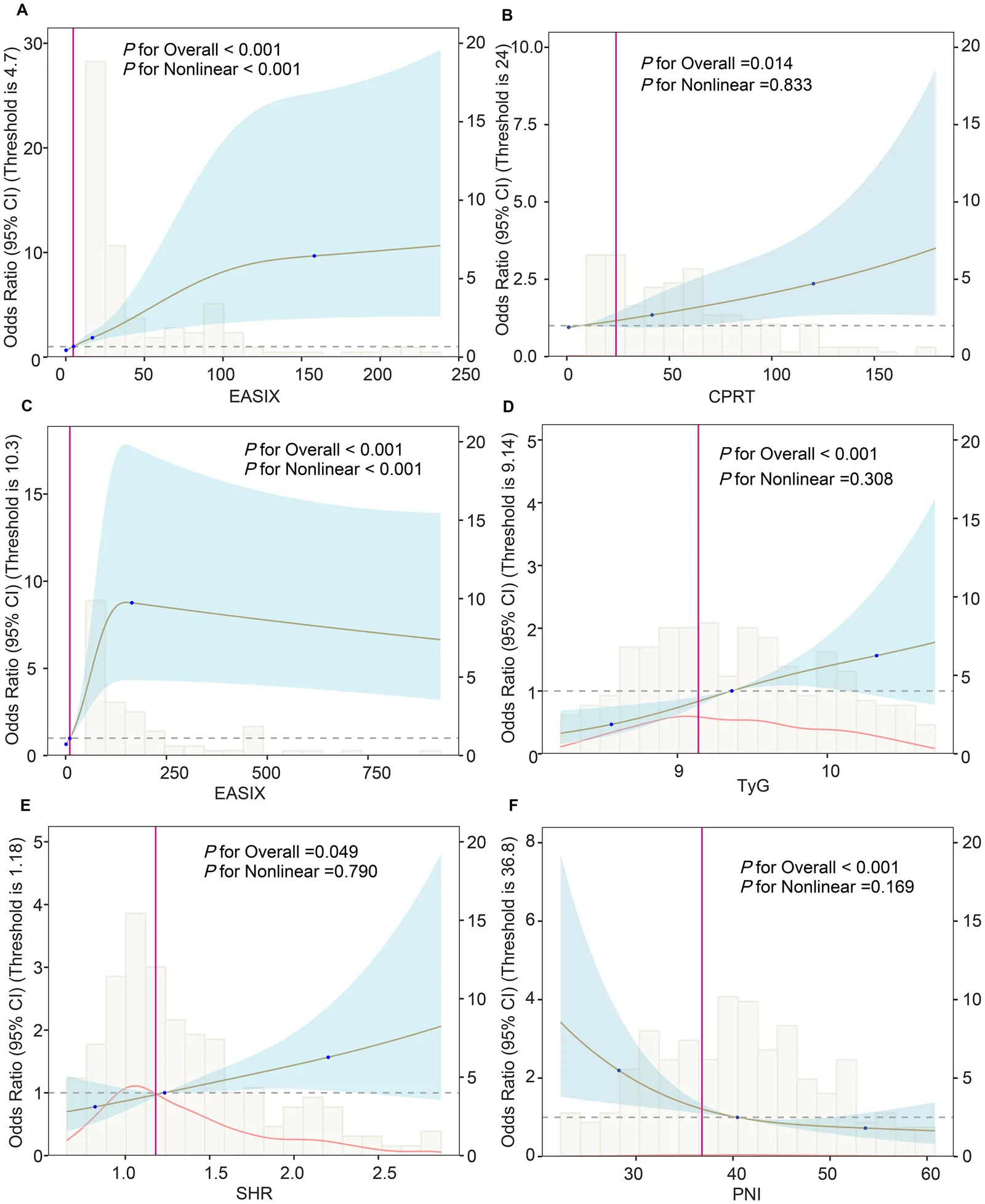
Potential nonlinear association for the levels of predictors with ECMO-AKI risk measured in pre-ECMOAKI and on-ECMOAKI models by restricted cubic spline regression with optimal knots. **(A)** EASIX in pre-ECMOAKI model. **(B)** CPRT in pre-ECMOAKI model. **(C)** EASIX in on-ECMOAKI model. **(D)** TyG in on-ECMOAKI model. **(E)** SHR in on-ECMOAKI model. **(F)** PNI in on-ECMOAKI model.

Second, threshold effect analysis was performed to identify inflection point for CPRT (24 min), lactate (4.5 and 2.1 mmol/L in pre-ECMOAKI and on-ECMOAKI models, respectively), Cr (126 and 120 μmol/L in pre-ECMOAKI and on-ECMOAKI models, respectively), TP (53.3 g/L), TBI (20.2 and 21.3 μmol/L in pre-ECMOAKI and on-ECMOAKI models, respectively), ProCT (1.26 ng/mL), PT (15.2 and 13.5 s in pre-ECMOAKI and on-ECMOAKI models, respectively), EASIX (4.7 and 10.3 in pre-ECMOAKI and on-ECMOAKI models, respectively), MAP (73 mmHg), WBC (16.39 ×10^9^/L), SHR (1.18), PNI (36.8), TyG (9.14). In detail, when any predictor rose above or declined below its corresponding inflection threshold, the probability of developing ECMO-AKI increased significantly (**Supplementary Material 13**).

Third, paired tests were applied to assess alterations in predictor levels across the pre-ECMO and on-ECMO periods (**Figures 7A–G**). The results indicated that among patients with ECMO-AKI, lactate, TBI, PT, SHR, PNI, and EASIX changed significantly from pre-ECMO to on-ECMO. In contrast, these indicators exhibited no significant changes in non-AKI patients, with the exception of lactate. Serum Cr did not differ significantly over the two time points in either AKI or non-AKI patients.

**Figure 7:**
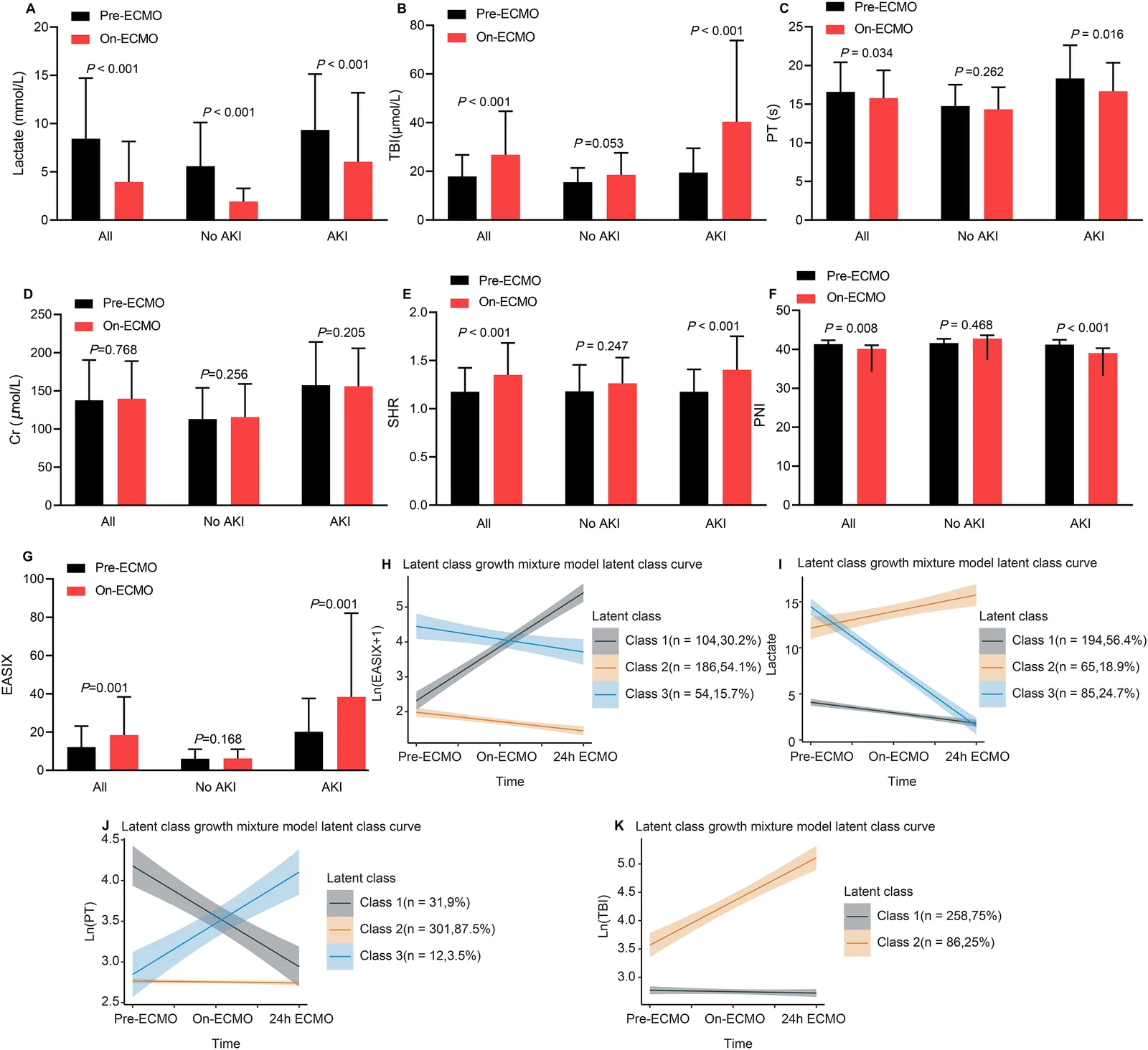
The dynamic trends and longitudinal trajectories of core predictive variables during the pre-ECMO and on-ECMO periods. **(A–G)** Paired tests were adopted to compare the changing trends of lactate **(A)**, TBI **(B)**, PT **(C)**, Cr **(D)**, SHR **(E)**, PNI **(F)**, and EASIX **(G)** between the pre-ECMO and on-ECMO stages among AKI, non-AKI and total populations. **(H–K)** The latent class growth mixture model (LCGMM) was applied to explore the associations between longitudinal EASIX (H, 3 classes), lactate (I, 3 classes), PT (J, 3 classes), and TBI (K, 2 classes) trajectories and the occurrence of ECMO-AKI. Owing to substantial data dispersion of EASIX, PT and TBI, natural log transformation was applied: ln(EASIX + 1), ln(PT), ln(TBI).

Fourth, LCGMM was used to examine links between longitudinal variable trajectories and ECMO-AKI development (**Figures 7H–K**). Based on the lower BIC, minimum subgroup proportion > 10% and mean posterior probabilities > 0.7, EASIX, lactate, and TBI were classified into 3, 3, and 2 classes, respectively (**Supplementary material 14**). PT was excluded for failing to meet the 10% subgroup threshold. For EASIX: Class 1 (30.23%, n=104), Class 2 (54.07%, n=186) and Class 3 (15.70%, n=54) had ECMO-AKI incidences of 74.04%, 46.77% and 66.67%, respectively, which were defined as the “low-rapid rise”, “low-slow decline” and “high-slow decline” phenotypes. The ECMO-AKI incidence was significantly higher in Class 1 compared with Class 2 (74.04% vs. 46.77%, Chi-square = 18.74, *P* < 0.001). For lactate: Class 1 (56.40%, n=194), Class 2 (18.90%, n=65) and Class 3 (24.71%, n=85) had ECMO-AKI incidences of 47.94%, 84.62% and 61.18%, respectively, which corresponded to the “low-slow decline”, “high-slow rise” and “high-slow decline” phenotypes. The ECMO-AKI incidence was significantly lower in Class 1 compared with Class 2 (47.94% vs. 84.62%, Chi-square = 23.31, *P* < 0.001). For TBI, Class 1 (75.00%, n=258) and Class 2 (25.00%, n=86) exhibited ECMO-AKI incidences of 49.61% and 83.72%, corresponding to the “low-stable” and “high-rapid rise” phenotypes. The ECMO-AKI incidence was significantly lower in Class 1 compared with Class 2 (49.61% vs. 83.72%, Chi-square = 28.32, *P* < 0.001).

## Discussion

ECMO-AKI represents a critical driver of mortality in ECMO-supported patients. Promptly identifying ECMO-supported individuals at elevated risk affords an opportunity for preemptive management, which is vital to reduce the incidence of ECMO-AKI. This study intends to systematically collect pre-ECMO and on-ECMO time-series data to develop staged dynamic ML models for ECMO-AKI prediction. Nine-feature pre-ECMOAKI and ten-feature on-ECMOAKI prediction models based on glmBoost + GBM algorithm combination achieved excellent discrimination, favorable calibration and reliable robustness. The two-staged ML models serve as an accurate, online-calculable, interpretable and accessible diagnostic tool for ECMO-AKI. The concordance predictive framework integrating pre-ECMOAKI and on-ECMOAKI models further enhances predictive accuracy and extends the actionable time window for clinical prophylaxis and intervention of ECMO-AKI.

To date, only a small retrospective cohort study (n = 139) has developed a nomogram incorporating Cr, lactate, the ECMO model and uric acid for ECMO-AKI prediction using on-ECMO data (Wang et al., 2022). This existing model has several prominent limitations: 1) The insufficient sample size undermines model reliability, triggering overfitting and yielding overoptimistic predictive performance; 2) The conventional modeling approach suffers from reduced predictive power due to failing to capture nonlinear variable relationships and higher overfitting risks with limited samples than ML algorithms. 3) Developed using only static clinical variables, the model cannot adequately reflect the dynamic changes of renal function, thus lacking real-time adaptability. 4) Absence of transparent interpretability and accessible online risk calculators restricts its clinical translation and application. Thus, with an adequate sample size, our study firstly developed a time-phased predictive architecture containing two independent models using multiple classic ML algorithms, which allows real-time online evaluation of ECMO-AKI risk and facilitates early and accurate identification of ECMO-AKI.

To guarantee favorable accuracy, robust stability and clear interpretability of the ECMO-AKI model, we carried out a series of optimized strategies in our research. First, univariate screening and Boruta algorithm-based variable selection were conducted to remove redundant variables and yield a concise, refined variable subset for the ECMO-AKI model’s practical implementation (Kumar et al., 2025). Finally, 12 and 16 variables were identified in the pre-ECMOAKI and on-ECMOAKI models, respectively. Second, the modeling framework for ECMO-AKI was proposed to enhance the model’s reliability, generalizability and stability. Prior studies usually selected modeling algorithms subjectively based on researchers’ personal experience and preference (Greener et al., 2022). To address this limitation, the ECMO-AKI prediction framework incorporated 12 ML algorithms to generate multiple algorithm combinations, facilitating the identification of the optimal modeling strategy. Overfitting commonly plagues biomedical ML models, with strong derivation-cohort performance failing to translate to validation data (Sheppert, 2026). The top three AUC-ranked pre-ECMO models and top four AUC-ranked on-ECMO models suffered obvious overfitting, while “glmBoost+GBM” did not. Balancing discriminative performance, goodness-of-fit, and model parsimony, the “glmBoost+GBM” model was determined as the optimal ECMO-AKI prediction model. Third, ML algorithms are often criticized as “black-box” tools owing to poor interpretability of their internal prediction mechanisms (Bienefeld et al., 2023). To unpack the opaque nature of our ECMO-AKI predictive models, we adopted SHapley Additive exPlanations (SHAP) analysis (He et al., 2025). This approach enables global interpretation of overall model behavior as well as individualized risk explanations by quantifying variable contributions for each patient’s ECMO-AKI prediction. In pre-ECMOAKI and on-ECMOAKI models, SHAP analysis identified EASIX, CPRT, lactate, TBI, PT, SHR, PNI, TyG as the top predictors for the ECMO-AKI model. These results were consistent with outputs from the Boruta algorithm, which also underscored them as core predictive variables. Fourth, an online computing platform was developed based on the “glmBoost+GBM” algorithms for pre-ECMOAKI and on-ECMOAKI models to enable real-time individual risk computation. Such a web-based patient-level predictive tool is freely accessible to clinicians, enabling them to combine model predictions with clinical experience to promptly identify patients at high risk of ECMO-AKI.

ECMO-AKI prediction faces an inherent trade-off between actionable early warning and predictive accuracy. Pre-ECMO models forecast risk prior to support but perform poorly by ignoring ECMO-related effects. Post-ECMO models yield better accuracy using post-initiation clinical data yet offer little utility for early ECMO-AKI prevention and intervention. To reconcile accuracy and actionable intervention windows, we proposed a predictive architecture incorporating two time-phased models (the pre-ECMOAKI and on-ECMOAKI models), which facilitates early and precise ECMO-AKI risk stratification. These findings highlight its ability to strengthen clinical reliability for ECMO-AKI risk evaluation. For consensus high-risk patients, clinicians should implement hemodynamic optimization, avoid nephrotoxic agents, control inflammation and infection, limit contrast medium exposure, and arrange early planning for RRT to mitigate ECMO-AKI and deliver timely intervention (Gist et al., 2026). For consensus low-risk patients, clinicians may reduce intensive surveillance of AKI, optimize the treatment plan combined with early rehabilitation interventions, and facilitate timely weaning from ECMO. For patients with inconsistent predictions between the two models, intensified monitoring and prompt protective strategies should be applied to enable early recognition and management of ECMO-AKI (Kellum et al., 2021). Though the precise mechanism of this synergism is yet to be clarified, the two models likely deliver complementary risk information to jointly improve prediction efficiency. Features incorporated in pre-ECMO models, such as gender, Cr and CPRT, reflect genetic susceptibility, baseline renal function and renal ischemic-hypoxic injury, providing essential insights into renal recovery potential. While, the on-ECMO model captured the pathophysiological responses following ECMO initiation, with features such as EASIX, TyG, and SHR. When the above risk factors coexist simultaneously, the pathological trajectory of renal injury becomes more predictable from a pathophysiological perspective. In conclusion, the model incorporates multi-timepoint predictive outputs for ECMO-AKI risk management, allowing clinicians to conduct more precise risk stratification and deliver individualized interventions.

To facilitate the clinical translation of these predictive variables incorporated in the pre-ECMOAKI and on-ECMOAKI models, RCS regression, threshold effect analysis, pairwise comparison tests, and LCGMM were employed to comprehensively comprehensively the relationships between these predictors and ECMO-AKI risk from static and dynamic dimensions. These predictive indicators correspond to diverse pathophysiological processes involved in AKI development (Chen J. et al., 2026), encompassing genetic susceptibility (gender), renal hypoperfusion (lactate and MAP), renal ischemia-hypoxia injury (CPRT), metabolic disturbance and toxin accumulation (Cr, TBI, and TyG), malnutrition status (TP and PNI), systemic infection and inflammatory responses (ProCT and WBC), coagulation disorders (PT), as well as endothelial activation and oxidative stress (EASXI and SHR). Therefore, the effective integration of these multi-mechanism predictors enables precise prediction of the risk of ECMO-AKI. EASIX quantifies endothelial activation and systemic stress (Jourde-Chiche et al., 2019). Endothelial damage and oxidative stress are established AKI risk factors that impair renal recovery, and prior work has validated EASIX as a critical AKI predictor in malignancy patients (Deng et al., 2024). Following ECMO initiation, extensive blood contact with artificial circuit surfaces and non-physiological blood shear stress trigger severe stress responses and renal microvascular endothelial damage, ultimately culminating in AKI (Sun et al., 2025). Feature importance analysis revealed EASIX was one of the most important predictors in both pre-ECMO and on-ECMO AKI models. This simple EASIX exhibits superior diagnostic performance, outperforming Cr in diagnosing ECMO-AKI, with predictive power matching APACHE II and SOFA-2. Paired tests uncovered before and during ECMO: EASIX rose sharply in AKI patients, whereas no significant alterations were observed in non-AKI patients. The EASIX trajectory subtype with “low-rapid rise” indicated a high risk of subsequent ECMO-AKI. Overall, EASIX can serve as a convenient surrogate marker for predicting ECMO-AKI. Clinicians should pay close attention to patients with drastically elevated EASIX after ECMO initiation, given their high risk of developing ECMO-AKI. Inflection point of modifiable predictors generated through threshold effect analysis may influence several clinical actions, such as when pre-ECMO lactate > 4.5 mmol/L, on-ECMO lactate > 2.1 mmol/L, or on-ECMO MAP < 73 mmHg, this warns the need for hemodynamic optimization to improve perfusion pressure; when pre-ECMO Cr > 126 μmol/L, on-ECMO Cr > 120 μmol/L, pre-ECMO TBI > 20.2 μmol/L or on-ECMO TBI > 21.3 μmol/L, this cues the need for early planning for RRT; when pre-ECMO TP < 53.3 g/L or on-ECMO PNI < 36.8, this warns the requirement for nutritional support (adequate energy and protein delivery); when pre-ECMO ProCT > 1.26 ng/mL or on-ECMO WBC > 16.39 ×10^9^/L, this cues the need for rational antibiotic administration for infection control; when on-ECMO TyG > 9.14, this warns the requirement for intensive glycemic and lipid control; when pre-ECMO EASIX > 4.7 or on-ECMO EASIX > 10.3, this cues the need for avoiding excessive vasoconstrictors-induced endothelial ischemic injury and antioxidant supplementation with vitamin C and E. Given that these results have not been implemented in clinical practice, their practical value remains unclear. Further prospective clinical trials are required to validate these findings.

Although we have endeavored to conduct this study with maximum rigor and comprehensiveness, several limitations remain to be acknowledged. First, given its single-center retrospective design with limited sample size, this study is devoid of external and prospective validation, resulting in limited generalizability, degree of overfitting risk and unavoidable inherent biases. While the model achieved satisfactory cross-validation performance, it cannot be generalized for routine clinical utilization until validated using large-scale, prospective, multicenter cohorts. Second, model selection in this study was not implemented within a nested resampling loop, where the test-set remains untouched until model finalization. Nested cross-validation represents the gold-standard to mitigate model-selection bias and winner’s-curse effects. However, nested resampling was computationally infeasible here due to heavy computational costs from ten multiply-imputed datasets. Future work should adopt nested cross-validation to further validate our findings. Third, because death constitutes a competing risk for ECMO-AKI, sensitivity analysis excluding early deaths (< 48 h) supports the robustness of our findings. Nevertheless, prospective validation using time-to-event endpoints with Fine-Gray subdistribution hazard modeling is warranted. Fourth, although this analytical framework enables reliable risk stratification, it cannot offer direct guidance for individualized targeted interventions. Future investigations should integrate predictive modeling with tailored therapeutic measures—namely, hemodynamic stabilization optimization and goal-directed fluid management—to prevent kidney injury. Fifth, predictive models fundamentally capture correlations instead of causal relationships, potentially limiting their reliability and utility in clinical decision workflows. Incorporating causal inference approaches such as causal forests is therefore critical to untangle the complex interactions between predictors and ECMO-AKI incidence. Sixth, the clinical efficacy of this model—namely, whether its incorporation into routine clinical workflows yields tangible improvements in patient outcomes—remains to be verified through prospective interventional trials. Seventh, novel renal injury biomarkers such as KIM-1, NGAL, and TIMP-2 × IGFBP7 were not included in the present analysis due to insufficient routine clinical data accessibility; nevertheless, our model architecture readily allows for their incorporation in follow-up research. Eighth, although we have thoroughly characterized the correlations between the included predictors (e.g., sex, CPRT, EASIX, TyG and TBI) and ECMO-AKI, the underlying physiological mechanisms through which these factors drive the development of ECMO-AKI remain poorly understood. Further mechanistic laboratory investigations are warranted to unravel the pathogenic pathways of ECMO-AKI in future work. Ninth, threshold effect analyses identified inflection points for each predictor. These findings are intended to generate hypotheses rather than provide definitive interventional guidance. As no interventional trials have yet validated these cutoffs, the real clinical utility of the derived predictor inflection points remains undetermined.

## Conclusion

Two-stage dynamic predictive models (pre-ECMOAKI and on-ECMOAKI) were built via glmBoost combined with GBM. They function as precise, user-friendly and interpretable diagnostic tools for identifying ECMO-AKI. Consistency assessment of predictive outputs from the two models demonstrates that this framework with high class-specific precision enables early risk stratification, exhibiting promise for personalized clinical management.

## Data Availability

The original contributions presented in the study are included in the article/**Supplementary Material**. Further inquiries can be directed to the corresponding authors.

## Supplementary material

Supplementary Figure 1 A total of 66 ML algorithm combinations of prediction models and further calculated the area under curve (AUC) of each model in derivation and validation cohorts for pre-ECMOAKI model.

Supplementary Figure 2 A total of 91 ML algorithm combinations of prediction models and further calculated the area under curve (AUC) of each model in derivation and validation cohorts for on-ECMOAKI model.

Supplementary Figure 3 5-fold cross-validation was applied to evaluate the reliability and generalization ability of pre-ECMOAKI model across 5 distinct cohorts. **(A)** ROC analysis. **(B)** PR analysis. **(C)** Calibration curves analysis. **(D)** DCA analysis.

Supplementary Figure 4 5-fold cross-validation was applied to evaluate the reliability and generalization ability of on-ECMOAKI model across 5 distinct cohorts. **(A)** ROC analysis. **(B)** PR analysis. **(C)** Calibration curves analysis. **(D)** DCA analysis.

Supplementary Figure 5 Bootstrap resampling was applied to evaluate the reliability and generalization ability of pre-ECMOAKI model across 5 distinct cohorts. **(A)** ROC analysis. **(B)** PR analysis. **(C**) Calibration curves analysis. **(D)** DCA analysis.

Supplementary Figure 6 Bootstrap resampling was applied to evaluate the reliability and generalization ability of on-ECMOAKI model across 5 distinct cohorts. **(A)** ROC analysis. **(B)** PR analysis. **(C)** Calibration curves analysis. **(D)** DCA analysis.

Supplementary Figure 7 One-way SHAP dependence plot of the important predictors in pre-ECMOAKI model. **(A)** Gender. **(B)** CPRT. **(C)** Lactate. **(D)** ProCT. **(E)** TP. **(F)** TBI. **(G)** PT. **(H)** Cr. **(I)** EASIX. Each dependence plot shows how a single feature affects the output of the prediction model, and each dot represents a single patient. Specifically, the values of the predictor are represented by the x-axis, and its SHAP values are represented by the y-axis.

Supplementary Figure 8 One-way SHAP dependence plot of the important predictors in on-ECMOAKI model. **(A)** MAP. **(B)** WBC. **(C)** Lactate. **(D)** TBI. **(E)** PT. **(F)** Cr. **(G)** SHR. **(H)** TyG. **(I)** PNI. **(J)** EASIX. Each dependence plot shows how a single feature affects the output of the prediction model, and each dot represents a single patient. Specifically, the values of the predictor are represented by the x-axis, and its SHAP values are represented by the y-axis.

Supplementary Figure 9 Potential nonlinear association for the levels of predictors with ECMO-AKI risk measured in pre-ECMOAKI model by restricted cubic spline regression with optimal knots. **(A)** Lactate. **(B)** ProCT. **(C)** TP. **(D)** TBI. **(E)** PT. **(F)** Cr.

Supplementary Figure 10 Potential nonlinear association for the levels of predictors with ECMO-AKI risk measured in on-ECMOAKI model by restricted cubic spline regression with optimal knots. **(A)** MAP. **(B)** Lactate. **(C)** WBC. **(D)** TBI. **(E)** PT. **(F)** Cr.

## Glossary

AKI: acute kidney injury
ECMO: extracorporeal membrane oxygenation
ICU: intensive care unit
ML: machine learning
RCS: restricted cubic spline
SHAP: Shapley Additive Explanations
CI: confidence interval
OR: odds ratio
SMD: standardized mean differences
ROC: receiver operating characteristic
AUC: area under curve
Enet: elastic network
LASSO: least absolute shrinkage and selection operator
RF: random forest
GBM: gradient boosting machine
SuperPC: supervised principal components
plsRglm: partial least squares regression for generalized linear models, SVM, support vector machine
DCA: decision curve analysis
LDA: linear discriminant analysis
XGBoost: eXtreme Gradient Boosting
GBM: gradient boosting machine
STROBE: Strengthening the Reporting of Observational Studies in Epidemiology
TRIPOD: Transparent Reporting of a Multivariable Prediction Model for Individual Prognosis or Diagnosis
EASIX: endothelial activation and stress index
CPRT: cardiopulmonary resuscitation time
TBI: total bilirubin
LCGMM: latent class growth mixture model
UO: urine output
KDIGO: Kidney Disease, Improving Global Outcomes
ELSO: Extracorporeal Life Support Organization
CKD: chronic kidney disease
RRT: renal replacement therapy
MCC: Matthews correlation coefficient
FNR: false negative rate
FPR: false positive rate
SD: standard deviation
VA: venoarterial
VV: venovenous
VAV: venoarterial-venous
EMRs: electronic medical records
AI: artificial intelligence
EPV: events-per-variable
TyG: triglyceride-glucose
BG: blood glucose
SHR: stress hyperglycemia ratio
PNI: prognostic nutritional index
MCAR: missing completely at random
MICE: multiple imputation with chained equations
VIF: variance inflation factor
PR: precision-recall
BIC: Bayesian Information Criterion
IQR: interquartile range
SOFA: sequential organ failure assessment
APACHE II: acute physiology and chronic health evaluation II
MAP: mean arterial pressure
WBC: white blood cell
PLT: platelet
Cr: creatinine
LDH: lactate dehydrogenase
TBI: total bilirubin
DBI: direct bilirubin
IBI: indirect bilirubin
TP: total protein
PT: prothrombin time
APTT: activation partial thromboplastin time
ProCT: procalcitonin
CK-MB: creatine kinase isoenzymes
HCT: hematocrit
LOS: length of stay
MVT: mechanical ventilation time
ECMOT: extracorporeal membrane oxygenation time
SD: standard deviation

## References

[B19] Ali J. Vuylsteke A. (2019). Extracorporeal membrane oxygenation: Indications, technique and contemporary outcomes. Heart 105, 1437–1443. doi: 10.1136/heartjnl-2017-311928 31040171

[B30] Bienefeld N. Boss J. M. Lüthy R. Brodbeck D. Azzati J. Blaser M. . (2023). Solving the explainable AI conundrum by bridging clinicians' needs and developers' goals. NPJ Digit Med. 6, 94. doi: 10.1038/s41746-023-00837-4 37217779 PMC10202353

[B33] Chen J. Xu X. Cai Y. Zhang Q. Wang X. Zhang D. (2026). Acute kidney injury over the past decade: From definition evolution to pathogenesis insights and innovative therapeutic strategies. Cell. Mol. Life Sci. 83, 156. doi: 10.1007/s00018-026-06104-5 41792449 PMC13003055

[B10] Chen W. Pei M. Chen C. Zhu R. Wang B. Shi L. . (2023). Independent risk factors of acute kidney injury among patients receiving extracorporeal membrane oxygenation. BMC Nephrol. 24, 81. doi: 10.1186/s12882-023-03112-6 36997848 PMC10064517

[B15] Chen Z. Zeng Z. Liu G. Su Y. Liu C. Zhong Y. . (2026). Development and internal validation of an interpretable machine learning model to predict coagulopathy following extracorporeal membrane oxygenation: A retrospective multicenter study. Scand. J. Trauma Resusc Emerg. Med. 34, 45. doi: 10.1186/s13049-026-01564-x 41606638 PMC12924354

[B18] Collins G. S. Moons K. G. M. Dhiman P. Riley R. D. Beam A. L. Van Calster B. . (2024). TRIPOD+AI statement: Updated guidance for reporting clinical prediction models that use regression or machine learning methods. BMJ 385, e078378. doi: 10.1136/bmj-2023-078378 38626948 PMC11019967

[B17] Cuschieri S. (2019). The STROBE guidelines. Saudi J. Anaesth. 13, S31–S34. doi: 10.4103/sja.SJA_543_18 30930717 PMC6398292

[B21] Deng F. Lin J. Lin H. (2024). Association between EASIX and acute kidney injury in critically ill cancer patients. BMC Nephrol. 25, 453. doi: 10.1186/s12882-024-03887-2 39696015 PMC11653550

[B3] Feistritzer H. J. Zeymer U. Ouarrak T. Akin I. Rassaf T. Lehmann R. . (2025). Different mechanical circulatory support strategies for infarct-related cardiogenic shock: A subanalysis of the ECLS-SHOCK trial. JACC Cardiovasc. Interv. 18, 691–701. doi: 10.1016/j.jcin.2024.12.010 40139846

[B14] Ge X. Chen W. Shi J. Zhang J. Tai H. Zhang Y. . (2025). Prediction of moderate-to-severe sepsis-associated acute kidney injury using a dual-timepoint machine learning model: Development, multiregional validation, and clinical deployment study. J. Med. Internet Res. 27, e73840. doi: 10.2196/73840 41027023 PMC12521856

[B31] Gist K. M. Selewski D. T. Kashani K. B. Akcan-Arikan A. Annich G. Antonini M. V. . (2026). Extracorporeal membrane oxygenation, acute kidney injury, fluid balance, and continuous renal replacement therapy: Acute Disease Quality Initiative (ADQI) and Extracorporeal Life Support Organization (ELSO) joint consensus conference. Intensive Care Med. 52, 1175–1195. doi: 10.1007/s00134-026-08440-3 42113209 PMC13269464

[B6] Göcze I. Jauch D. Götz M. Kennedy P. Jung B. Zeman F. . (2018). Biomarker-guided intervention to prevent acute kidney injury after major surgery: The prospective randomized BigpAK study. Ann. Surg. 267, 1013–1020. doi: 10.1097/SLA.0000000000002485 28857811

[B28] Greener J. G. Kandathil S. M. Moffat L. Jones D. T. (2022). A guide to machine learning for biologists. Nat. Rev. Mol. Cell Biol. 23, 40–55. doi: 10.1038/s41580-021-00407-0 34518686

[B4] Guru P. K. Balasubramanian P. Ghimire M. Bohman J. K.K. Seelhammer T. G. Kashani K. B. . (2024). Acute kidney injury in patients before and after extracorporeal membrane oxygenation (ECMO) - Retrospective longitudinal analysis of the hospital outcomes. J. Crit. Care 81, 154528. doi: 10.1016/j.jcrc.2024.154528 38295627

[B1] Hadaya J. Benharash P. (2020). Extracorporeal membrane oxygenation. JAMA 323, 2536. doi: 10.1001/jama.2020.9148 32463441

[B25] He J. Wang X. Zhu P. Wang X. Zhang Y. Zhao J. . (2025). Identification and validation of an explainable early-stage chronic kidney disease prediction model: A multicenter retrospective study. EClinicalMedicine 84, 103286. doi: 10.1016/j.eclinm.2025.103286 40567347 PMC12192353

[B34] Jourde-Chiche N. Fakhouri F. Dou L. Bellien J. Burtey S. Frimat M. . (2019). Endothelium structure and function in kidney health and disease. Nat. Rev. Nephrol. 15, 87–108. doi: 10.1038/s41581-018-0098-z 30607032

[B32] Kellum J. A. Romagnani P. Ashuntantang G. Ronco C. Zarbock A. Anders H. J. (2021). Acute kidney injury. Nat. Rev. Dis. Primers 7, 52. doi: 10.1038/s41572-021-00284-z 34267223

[B7] Kidney Disease: Improving Global Outcomes (KDIGO) Acute Kidney Injury Work Group (2012). KDIGO clinical practice guideline for acute kidney injury. Kidney Int. Suppl. 2, 1–138. doi: 10.1038/kisup.2012.1

[B27] Kumar S. Varshney S. Jain U. Johri P. Almazyad A. S. Mohamed A. W. (2025). EODA: A three-stage efficient outlier detection approach using Boruta-RF feature selection and enhanced KNN-based clustering algorithm. PloS One 20, e0322738. doi: 10.1371/journal.pone.0322738 40445940 PMC12124576

[B16] Lapp L. Roper M. Kavanagh K. Bouamrane M. M. Schraag S. (2023). Dynamic prediction of patient outcomes in the intensive care unit: A scoping review of the state-of-the-art. J. Intensive Care Med. 38, 575–591. doi: 10.1177/08850666231166349 37016893 PMC10302367

[B12] Lepère V. Duceau B. Lebreton G. Bombled C. Dujardin O. Boccara L. . (2020). Risk factors for developing severe acute kidney injury in adult patients with refractory postcardiotomy cardiogenic shock receiving venoarterial extracorporeal membrane oxygenation. Crit. Care Med. 48, e715–e721. doi: 10.1097/CCM.0000000000004433 32697513

[B23] Li L. Ding L. Zheng L. Wu L. Hu Z. Liu L. . (2024). Relationship between stress hyperglycemia ratio and acute kidney injury in patients with congestive heart failure. Cardiovasc. Diabetol. 23, 29. doi: 10.1186/s12933-023-02105-x 38218835 PMC10787441

[B24] Li Z. Xie M. Wu H. Wang T. Huang S. Cheng J. (2026). Multicenter evaluation of prognostic nutritional index and systemic immune-inflammation index in predicting mortality among critically ill cardiovascular and cerebrovascular patients with varied glucose metabolism: A machine learning-based cohort study. Front. Nutr. 13, 1703589. doi: 10.3389/fnut.2026.1703589 41710272 PMC12909231

[B11] Mou Z. Guan T. Chen L. (2022). Risk factors of acute kidney injury in ECMO patients: A systematic review and meta-analysis. J. Intensive Care Med. 37, 267–277. doi: 10.1177/08850666211003485 33761767

[B9] Ostermann M. Lumlertgul N. (2021). Acute kidney injury in ECMO patients. Crit. Care 25, 313. doi: 10.1186/s13054-021-03676-5 34461966 PMC8405346

[B20] Ranzani O. T. Singer M. Salluh J. I. F. Shankar-Hari M. Pilcher D. Berger-Estilita J. . (2025). Development and validation of the Sequential Organ Failure Assessment (SOFA)-2 score. JAMA 334, 2090–2103. doi: 10.1001/jama.2025.20516 41159833 PMC12573119

[B8] Ronco C. Bellomo R. Kellum J. A. (2019). Acute kidney injury. Lancet 394, 1949–1964. doi: 10.1016/S0140-6736(19)32563-2 31777389

[B29] Sheppert A. P. (2026). Techniques for mitigating overfitting in machine learning: A comprehensive review, taxonomy, and practical guide. Front. Artif. Intell. 9, 1794271. doi: 10.3389/frai.2026.1794271 41953863 PMC13053638

[B36] Sun K. Yao C. Xu G. Wang J. Shou S. Jin H. (2025). Research progress on the pathogenesis of AKI complicated by ECMO. Clin. Exp. Nephrol. 29, 10–20. doi: 10.1007/s10157-024-02559-7 39340702 PMC11807062

[B2] Tonna J. E. Boonstra P. S. MacLaren G. Paden M. Brodie D. Anders M. . (2024). Extracorporeal life support organization registry international report 2022: 100,000 survivors. ASAIO J. 70, 131–143. doi: 10.1097/MAT.0000000000002128 38181413 PMC10962646

[B13] van de Sande D. van Genderen M. E. Huiskens J. Gommers D. van Bommel J. (2021). Moving from bytes to bedside: A systematic review on the use of artificial intelligence in the intensive care unit. Intensive Care Med. 47, 750–760. doi: 10.1007/s00134-021-06446-7 34089064 PMC8178026

[B26] Wang L. Chen L. Ni H. Deng H. Chen K. Wang H. (2022). Development of an acute kidney injury risk prediction model for patients undergoing extracorporeal membrane oxygenation. Heliyon 8, e12585. doi: 10.1016/j.heliyon.2022.e12585 36643308 PMC9834743

[B22] Yang Z. Gong H. Kan F. Ji N. (2023). Association between the triglyceride glucose (TyG) index and the risk of acute kidney injury in critically ill patients with heart failure: Analysis of the MIMIC-IV database. Cardiovasc. Diabetol. 22, 232. doi: 10.1186/s12933-023-01971-9 37653418 PMC10472684

[B5] Zarbock A. Küllmar M. Ostermann M. Lucchese G. Baig K. Cennamo A. . (2021). Prevention of cardiac surgery-associated acute kidney injury by implementing the KDIGO guidelines in high-risk patients identified by biomarkers: The PrevAKI-multicenter randomized controlled trial. Anesth. Analg. 133, 292–302. doi: 10.1213/ANE.0000000000005458 33684086

